# Peptide RL‐QN15 Regulates Functions of Epidermal Stem Cells to Accelerate Skin Wound Regeneration via the FZD8/β‐Catenin Axis

**DOI:** 10.1002/EXP.20240090

**Published:** 2025-06-08

**Authors:** Yuansheng Li, Qiuye Jia, Naixin Liu, Saige Yin, Junyuan Wang, Yujing Ding, Yuliu Yang, Ying Peng, Zeqiong Ru, Shaoyang Zhang, Bu'er Qi, Jun Sun, Li He, Ying Wang, Kun Guo, Xinwang Yang

**Affiliations:** ^1^ Department of Anatomy and Histology & Embryology, Faculty of Basic Medical Science Kunming Medical University Kunming Yunnan China; ^2^ Key Laboratory of Chemistry in Ethnic Medicinal Resources & Key Laboratory of Natural Products Synthetic Biology of Ethnic Medicinal Endophytes, State Ethnic Affairs Commission & Ministry of Education, School of Ethnic Medicine Yunnan Minzu University Kunming Yunnan China; ^3^ Department of Restorative Dentistry, Faculty of Dental Medicine Hokkaido University Sapporo Japan; ^4^ Department of Dermatology First Affiliated Hospital of Kunming Medical University Kunming Yunnan China; ^5^ Yunnan Yunke Characteristic Plant Extraction Laboratory Co., Ltd. Kunming Yunnan China

**Keywords:** epidermal stem cells, FZD8 receptor, RL‐QN15, skin wound healing, Wnt/β‐catenin signaling pathway

## Abstract

The pursuit of developing groundbreaking pro‐regenerative therapies to expedite skin wound healing persists as a formidable challenge. Peptide RL‐QN15, emerges as a highly promising candidate for the first pro‐regenerative drug derived from amphibian skin, offering a glimmer of hope for innovative healing treatments. Yet, there is an urgent need for intensified research efforts to propel RL‐QN15 from a molecular entity to a viable drug candidate, particularly in unraveling the mechanisms underlying its exceptional pro‐healing efficacy. In the current research, our results revealed that RL‐QN15 significantly enhanced the proliferation, migration, stemness, and epithelial‐to‐mesenchymal transition of human epidermal stem cells (hESCs) through direct binding to the membrane frizzled 8 (FZD8) receptor. This interaction triggers the downstream Wnt/β‐catenin signaling pathway, leading to the up‐regulation of target genes MYC and CCND1. Furthermore, RL‐QN15 augmented the expression and secretion of matrix metalloproteinase‐3, which degrades E‐cadherin and activates the Wnt/β‐catenin pathway, thereby amplifying RL‐QN15's regulatory effects on hESCs. In summary, our findings have demonstrated that RL‐QN15 modulated the functions of ESCs to accelerate skin wound regeneration via the FZD8/β‐catenin axis. This research not only advances peptide RL‐QN15 from a molecular entity to a drug candidate by shedding light on the mechanisms involved with regulation of ESCs functions, but also presents compelling evidence implicating FZD8 as a novel therapeutic target for skin wound regeneration.

## Graphical abstract

Amphibian‐derived peptide RL‐QN15 activates the Wnt/β‐catenin signaling pathway by interacting with the FZD8 membrane receptor, promoting epidermal stem cells proliferation, migration, stemness, and EMT processes, thus promoting skin wound healing.

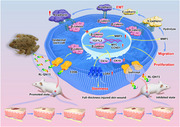

## 1 Introduction

The skin, being the largest organ of the human body, comprises intricate layers of epidermis, dermis, and subcutaneous tissue. It performs a pivotal role as a resilient barrier, safeguarding against mechanical trauma, infectious agents, and harmful ultraviolet radiation. Upon encountering injury, the skin initiates an intricate and sophisticated healing cascade. Nevertheless, this natural reparative process is often hindered by factors such as diabetes mellitus and the inevitable process of aging, leading to the development of chronic, recalcitrant wounds that defy healing. These lingering wounds pose a heightened risk of grave complications, encompassing the potential for limb amputation and life‐threatening sepsis, thereby imposing substantial stress on patients' quality of life and straining healthcare resources [1]. Consequently, the quest for discovering and developing innovative, pro‐regenerative therapeutic interventions aimed at accelerating skin wound healing remains a paramount challenge in the realm of regenerative medicine, necessitating relentless research efforts.

In recent years, pro‐healing peptides have garnered considerable attention within the realm of regenerative medicine, with an increasing number of such peptides being discovered and characterized from diverse sources, including microbes, plants, and animals [2]. Of particular interest are amphibian‐derived pro‐healing peptides distinguished by their exceptional properties of high activity, specificity, and safety [3]. Notably, approximately 30 pro‐healing peptides, exemplified by AH90, CW49, Cathelicidin‐OA1 and OM‐LV20, have been identified and reported from amphibian skin secretions [4, 5, 6, 7, 8, 9]. Our previous research endeavors have discovered a novel peptide, RL‐QN15, with the amino acid sequence ‘QNSYADLWCQFHYMC’, sourced from the skin secretions of the frog *Rana limnocharis*, and demonstrated that RL‐QN15 significantly accelerates skin wound healing at a relatively low concentration of nanomoles per liter, positioning it as one of the most potent natural pro‐healing molecules identified thus far [10]. Recent research advancements further underscore the promising potential of RL‐QN15‐based strategies in addressing skin wounds [11, 12, 13, 14, 15]. Despite the recognition of amphibian skin secretions as a treasure trove of peptides vital for drug development, with hundreds of peptides already identified, the translation of these discoveries into clinically viable drugs remains elusive, with no successful cases reported to date. Our studies suggest that RL‐QN15 holds substantial promise as the first pro‐regenerative drug derived from amphibian skin, offering great hope as an innovative pro‐healing therapy. Nevertheless, urgent and intensified efforts are warranted to advance RL‐QN15 from a molecular entity to a drug candidate, particularly in elucidating the underlying mechanisms that contribute to its remarkable pro‐healing efficacy.

The intricate nature of skin wound repair, spanning four intricately linked phases‐hemostasis, inflammation, proliferation, and remodeling [16], coupled with our limited comprehension, poses significant hurdles to the successful development of novel therapeutic agents for accelerating skin wound healing. Peptides have emerged as both a vital resource for drug discovery and a crucial tool molecule for deciphering the mechanisms of major human diseases. Their pivotal role in drug research and development, as well as in identifying and validating new drug targets, underscores peptides’ significance [17]. Recent advancements leveraging peptides as investigative tools have illuminated novel ligand/receptor interactions and epigenetic pathways in skin wound regeneration. Notably, employing peptides OA‐RD17 and Anersoinin‐W1 as probes, the TLR4 receptor has been unveiled as a promising therapeutic target for skin wound regeneration [18, 19]. Furthermore, groundbreaking findings using OA‐RD17, OA‐GP11d, and RL‐QN15 as probes have, for the first time, implicated miR‐4482, miR‐632, miR‐186‐5p, and miR‐663a in skin wound regeneration, positioning them as potential small nucleic acid therapeutics and drug targets [11, 18, 20, 24]. As the key functional cells of skin injury repair lie in epidermal stem cells (ESCs), which, upon wounding, become activated, initiating their migration and proliferation [25]. Extensive research underscores the necessity of ESCs proliferation and migration for epidermal reconstruction and functional restoration, thereby rendering them central to wound healing and tissue regeneration [26, 27, 28, 29, 30, 31, 32]. Consequently, ESCs have garnered significant attention and become a primary target in wound repair research [33]. However, the precise mechanism by which peptides exert their healing effects through modulating ESC function remains unexplored, making the investigation of RL‐QN15 as a pioneering case of utmost interest.

A myriad of factors, including regulatory elements spanning diverse niches, intricately modulate the functionality of epidermal stem cells (ESCs) [34, 35]. Foremost among them, the Wnt signaling pathway occupies a paramount position within stem cell niches, actively maintaining skin homeostasis, fostering growth, and orchestrating healing processes [36]. The Wnt/β‐catenin signaling pathway meticulously governs the differentiation and self‐renewal of ESCs, further enhancing tissue regeneration by regulating proliferation and epithelialization [37, 38]. Upon receptor activation, β‐catenin, a pivotal effector of the Wnt/β‐catenin signaling pathway, undergoes a crucial transformation. Inhibition of β‐catenin degradation triggers its accumulation in the cytoplasm, followed by translocation to the nucleus, where it interacts with TCF/LEF transcription factors to regulate genes such as c‐Myc and cyclin D1, thereby promoting skin wound healing [39, 40]. Moreover, β‐catenin interacts with the cytoplasmic domains of various cadherins, particularly E‐cadherin (CDH1) and N‐cadherin (CDH2) present in adherens junctions, which are crucial for cell adhesion during the epithelial‐mesenchymal transition (EMT) [41]. EMT, a vital step in wound healing, significantly contributes to tissue regeneration and the restoration of structural integrity at the injury site. Thus, the activation of the β‐catenin signaling pathway generally benefits skin wound healing. The Frizzled 8 (FZD8) receptor, a key player in the Frizzled protein family, holds a pivotal position in the Wnt signaling cascade, profoundly influencing neural development, cell differentiation, and cancer progression [42]. Although the activation of the FZD8 receptor leads to the activation of downstream β‐catenin, there is a conspicuous lack of reports elucidating the role of FZD8 in modulating ESC proliferation and migration to mediate skin wound healing. This gap in knowledge underscores the need for further investigation into the intricacies of these signaling pathways and their impact on skin regeneration.

In current research, we have conclusively demonstrated that the peptide RL‐QN15 directly binds to the FZD8 receptor residing in the membranes of ESCs. This interaction triggers the activation of the β‐catenin signaling pathway, which subsequently elicits a cascade of biological responses including proliferation, migration, enhancement of stemness, and facilitation of EMT in ESCs. These concerted effects ultimately expedite the process of skin wound healing. Our findings not only unravel a novel mechanism whereby peptides can promote skin wound repair by modulating the functional properties of ESCs but also, for the first time, propose FZD8 as a promising therapeutic target for enhancing skin wound regeneration. Furthermore, this research significantly elevates the status of RL‐QN15 from a mere molecular entity to a potential drug candidate, marking a significant step forward in the development of innovative treatments for skin wound regeneration.

## 2 Results

### 2.1 RL‐QN15 Significantly Promoted Proliferation, Migration, and Stemness of Human ESCs

**FIGURE 1:**
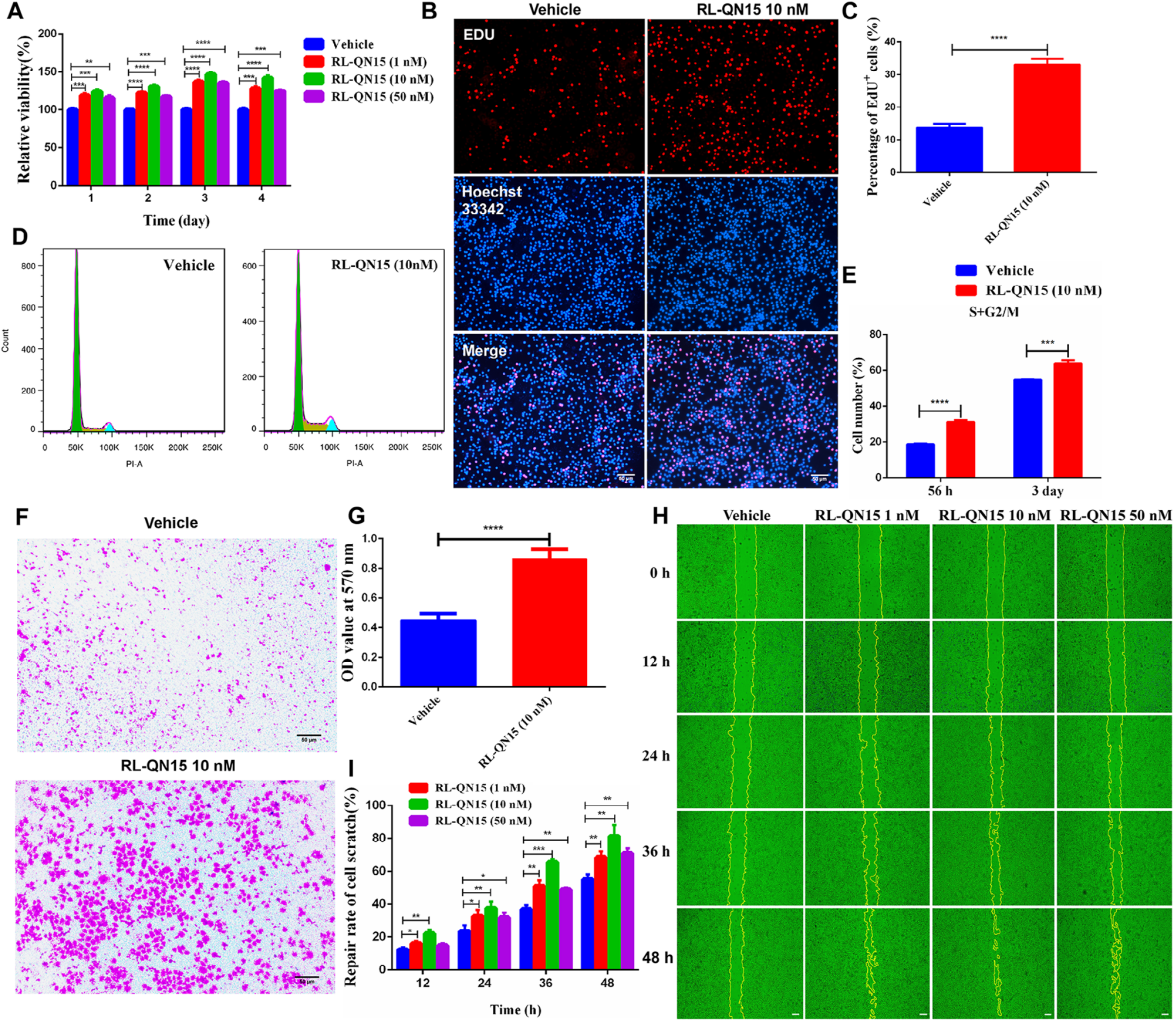
Comprehensive effects of RL‐QN15 on hESCs proliferation, migration, scratch repair, and cell cycle dynamics. (A) Quantitative assessment of the impact of three varying concentrations of RL‐QN15 on the proliferation and viability of hESCs (*n =* 5). (B,C) Visual representation and quantitative assessment of EdU‐positive cells (stained red) and nuclei (stained blue) in hESCs treated with 10 nM RL‐QN15 in EpiLife medium, respectively, (*n* = 3). Scale bar: 50 µm. (D) Representative flow cytometry images illustrating the alterations in the cell cycle phases of hESCs following treatment with 10 nM RL‐QN15 for 56 h. (E) Quantification of the percentage of hESCs residing in the S and G2/M phases of the cell cycle after treatment with 10 nM RL‐QN15 for 56 h and 3 days (*n* = 3). (F,G) Representative images and quantitative analysis of the enhanced migratory capacity of hESCs in response to 10 nM RL‐QN15 treatment, respectively (*n =* 5). Scale bar: 50 µm. (H,I) Micrographs and quantitative analysis depicting the efficacy of RL‐QN15 (1, 10, and 50 nM) in promoting scratch repair of hESCs (*n* = 5). The scratch repair rate was determined as the proportion of remaining gaps relative to a total area of the acellular region after the scratch. Scale bar: 100 µm. All data were expressed as mean ± SD, *p < 0.05, **p < 0.01, ***p < 0.001, and ****p < 0.0001.

Flow cytometry revealed the expression of specific molecular markers (CK19 and ITGB1) associated with human ESCs (hESCs) (Figure S1A,B) [43, 44], and hence confirmed the identity of the cultured cells as hESCs. ESCs are stimulated to proliferate during wound healing, chiefly through asymmetric division, thereby generating epidermal cells swiftly for wound closure and recovery of epidermal deficits. This proliferation of ESCs and keratinocytes on the extracellular matrix signifies the commencement of epithelialization, a pivotal phase in wound healing [45]. The impact of RL‐QN15 on the proliferation of hESCs was assessed using the MTS assay and our results demonstrated significant increases in hESCs proliferation at various RL‐QN15 concentrations on days 1, 2, 3, and 4, with the most notable enhancement observed at 10 nM (Figure 1A). An increase in the proportion of EDU‐positive hESCs treated with 10 nM RL‐QN15 further supported the potent proliferative effect of RL‐QN15 at this concentration (Figure 1B,C). To investigate the effect of RL‐QN15 on the cell cycle of hESCs, flow cytometry was utilized to quantify the DNA content of hESCs and determine cell population distribution across G0/G1, S, and G2/M phases. Results indicated that 10 nM RL‐QN15 significantly augmented the proportions of cells in the S and G2/M phases, particularly at 56 h (Figure 1D,E), suggesting that RL‐QN15 drives hESCs progression to S and G2/M phases for proliferation.

During wound healing, proliferating ESCs and their progeny migrate in a specific spatial arrangement to bridge the wound breach [46]. Depletion or impeded ESCs migration can significantly hinder wound healing [47]. Our transwell migration assay results clearly revealed that the 10 nM RL‐QN15 group had a significantly elevated quantity of migrating hESCs compared to the vehicle group (Figure 1F,G), implying that RL‐QN15 markedly enhanced hESCs migration. Correspondingly, the scratch assay results demonstrated that hESCs treated with 1, 10, and 50 nM RL‐QN15 noticeably accelerated scratch wound repair compared to the untreated vehicles, with the most pronounced effect observed at 10 nM (Figure 1H,I). To exclude the potential interference of cell proliferation on scratch repair, cells treated with mitomycin were utilized for the scratch repair assay. Our previous investigations have highlighted the significant impact of even 1 nM RL‐QN15 on HaCaT cells, underscoring the versatility of RL‐QN15 across varying concentrations in modulating epidermal cell behavior [10, 11, 13, 15]. Nevertheless, for hESCs, the most profound effect was observed at 10 nM RL‐QN15, prompting the selection of this concentration for all subsequent cellular experiments.

Furthermore, we delved into the expression patterns of genes associated with proliferation, migration, and stemness in RL‐QN15‐treated hESCs. Notably, a significant upregulation was observed in the proliferation‐related genes *MYC* and *CCND1* in hESCs exposed to 10 nM RL‐QN15 (Figure S2A). Additionally, we witnessed a marked increase in the expression of integrin molecules (*ITGA3*, *ITGA5*, *ITGA6* and *ITGB1*), vital for cell migration, along with members of the matrix metalloproteinases (MMPs) family (*MMP3*, *MMP15*), which contribute to tissue remodeling (Figure S2B,C). As an essential member of the MMP family, MMP3 is acclaimed for its contribution to tumor promotion by erosion of the basement membrane, augmenting cellular migration in mouse epithelial cells via EMT promotion [48]. The stemness features of hESCs, critical for regulating proliferation and migration, were also fortified, as evidenced by the upregulation of stemness marker genes *ITGB1*, *LGR4*, and *ITGA6* in hESCs treated with 10 nM RL‐QN15 (Figure S2C).

### 2.2 RL‐QN15 Enhanced hESCs Proliferation, Migration, Stemness, and EMT Through the β‐Catenin Signaling Pathway

**FIGURE 2:**
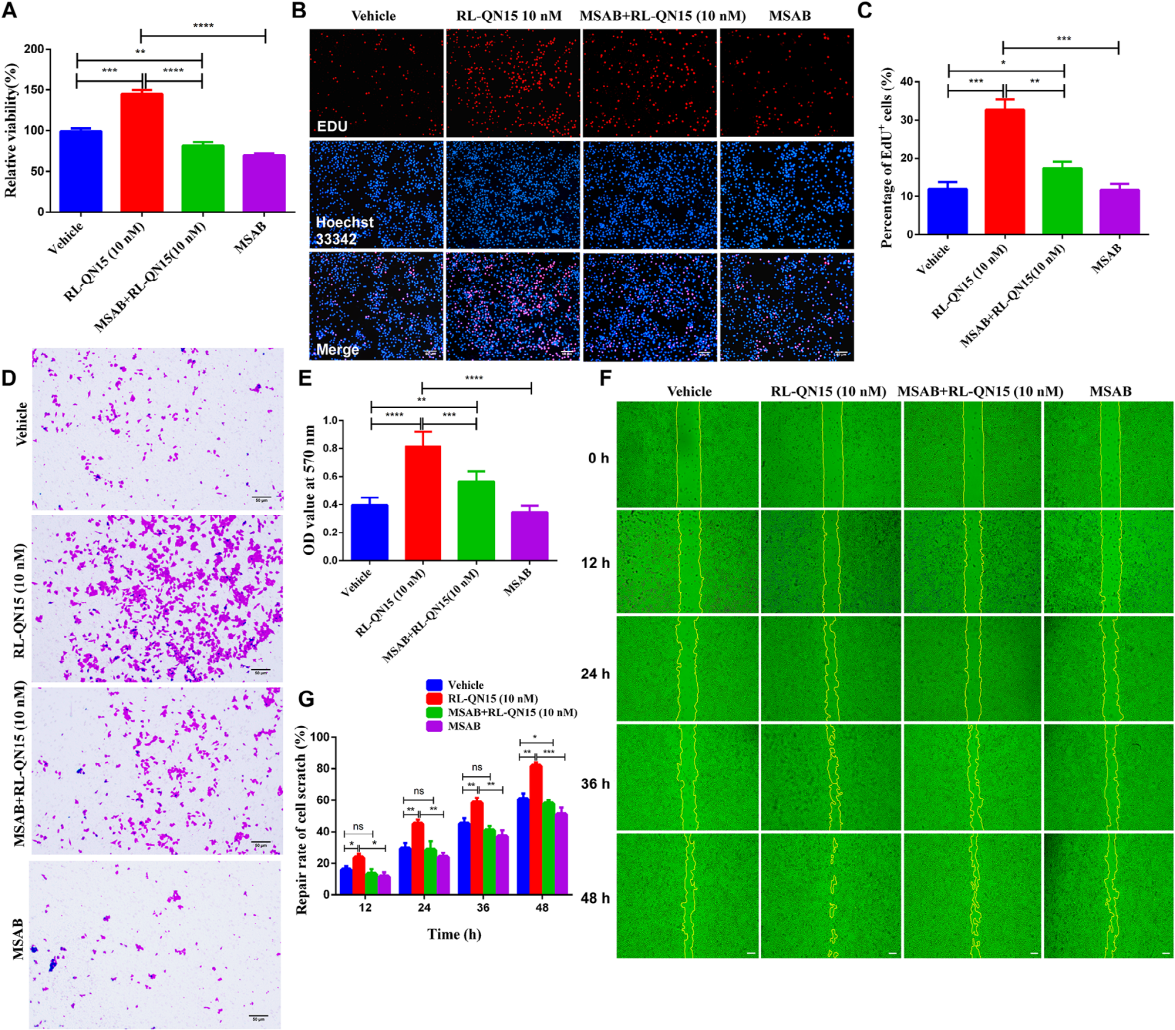
Impact of Wnt/β‐catenin pathway inhibitor MSAB on RL‐QN15‐induced hESCs proliferation, migration, and scratch repair. (A) Quantitative assessment of the impact of MSAB on the RL‐QN15‐induced hESCs proliferation and viability (*n =* 5). (B,C) Representative photographs and quantitative analysis of EdU positive cells (red) in hESCs treated with MSAB in 10 nM RL‐QN15, respectively (*n =* 3). Scale bar: 50 µm. (D,E) Representative images and quantitative analysis of hESCs migration following RL‐QN15 and MSAB treatment (*n =* 5). Scale bar: 50 µm. (F,G) Representative images and quantitative analysis showing the efficacy of RL‐QN15 on hESCs scratch repair in the presence/absence of MSAB (*n =* 5). Scale bar: 100 µm. All data were expressed as mean ± SD, *p < 0.05, **p < 0.01, ***p < 0.001, ****p < 0.0001. ns, No significance.

While previous evidence indicated that RL‐QN15 significantly enhances hESCs' proliferation, migration, and stemness, the underlying mechanism remains elusive. The Wnt/β‐catenin signaling pathway plays a pivotal role in skin development and wound repair by regulating the self‐renewal and differentiation of ESCs [37, 39, 49, 51], thereby facilitating tissue regeneration and remodeling [38], and we hypothesized that the capacity of RL‐QN15 to promote hESCs proliferation, migration and stemness may be mediated via regulating Wnt/β‐catenin signaling pathway. To test this hypothesis, we analyzed the expression levels of key Wnt/β‐catenin signaling pathway components in RL‐QN15‐treated hESCs. Our results revealed a significant upregulation of *CTNNB1*, a crucial pathway component, as well as notable increases in the expression of upstream receptor genes *FZD2* and *FZD8*, and co‐receptors *LRP5* and *LRP6*, along with downstream transcription factors *TCF7L1* and *TCF7L2* (Figure S2D). To further explore the role of RL‐QN15 in hESC proliferation, migration, and stemness via the Wnt/β‐catenin signaling pathway, we assessed if MSAB (methyl 3‐(4‐methylphenylsulfonamido)benzoate), a specificWnt/β‐catenin signaling pathway inhibitor [52], shows regulatory effects against hESCs treated with RL‐QN15. MTS assay results showed that MSAB significantly diminished the proliferative effects of RL‐QN15 on hESCs (Figure 2A). Similarly, the percentage of EDU‐positive cells decreased substantially when hESCs were treated with both RL‐QN15 and MSAB compared to RL‐QN15 alone (Figure 2B,C). The transwell assay revealed a substantial reduction in the number of migrated hESCs when MSAB was combined with RL‐QN15 compared to the RL‐QN15 treatment alone (Figure 2D,E). Additionally, the scratch assay demonstrated that MSAB considerably reduced the capacity for scratch repair in hESCs treated with RL‐QN15 at various time intervals compared to RL‐QN15 treatment alone (Figure 2F,G). These findings suggested that MSAB significantly inhibited the effects of RL‐QN15 on hESCs proliferation and migration, implying that RL‐QN15 regulated hESCs’ functions through the Wnt/β‐catenin signaling pathway.

**FIGURE 3:**
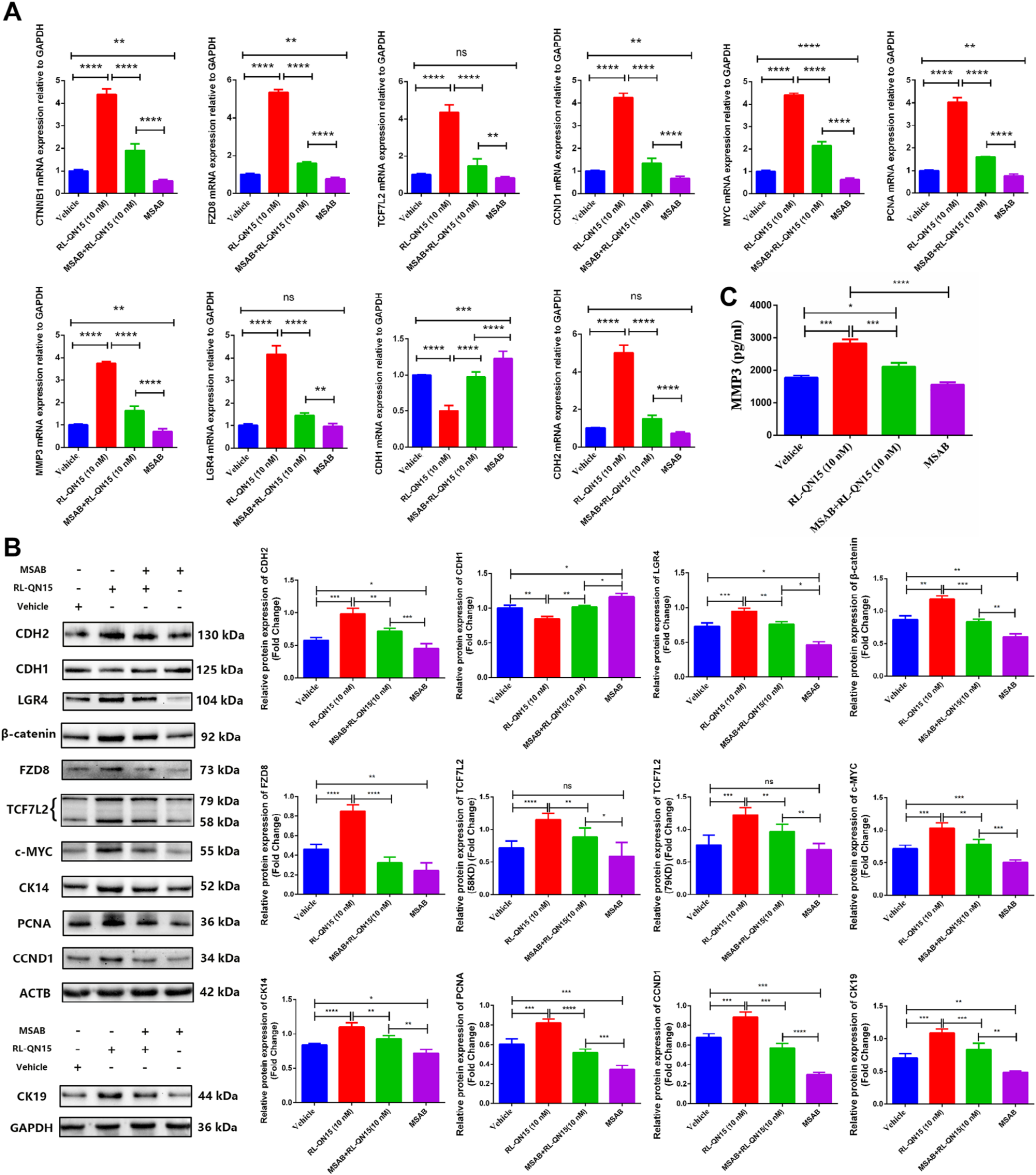
Effects of MSAB on Wnt/β‐catenin signaling pathway, proliferation, migration, stemness, and EMT‐related gene expression in RL‐QN15‐treated hESCs. (A) Effects of MSAB on the mRNA levels of proliferation‐related (*MYC*, *CCND1*, *PCNA*), migration‐related (*MMP3*), stemness‐related (*LGR4*), EMT‐related genes (*CDH1*, *CDH2*) and key members of the Wnt/β‐catenin signaling pathway (*CTNNB1, FZD8*, and *TCF7L2*) in 10 nM RL‐QN15‐treated hESCs. GAPDH expression level served as an internal reference. (B) Effects of MSAB on protein expression of proliferation‐related (CCND1, MYC, and PCNA), stemness‐related proteins (CK19, CK14, and LGR4), EMT‐related proteins (CDH1 and CDH2), and key members of the Wnt/β‐catenin signaling pathway (β‐catenin, FZD8, and TCF7L2) in 10 nM RL‐QN15‐treated hESCs. GAPDH and ACTB were used as internal references. (C) Effect of MSAB on secretion of MMP3 in 10 nM RL‐QN15‐treated hESCs. All data were expressed as mean ± SD (*n =* 3), *p < 0.05, **p < 0.01, ***p < 0.001, ****p < 0.0001, ns, no significance.

Our follow‐up experiments further substantiated the role of RL‐QN15 in modulating hESCs via the Wnt/β‐catenin signaling pathway. Initially, we observed that 10 nM RL‐QN15 significantly augmented mRNA levels of Wnt/β‐catenin signaling pathway components, including *CTNNB1*, *FZD2*, *FZD8*, *LRP5*, *LRP6*, *TCF7L1*, and *TCF7L2*, which were counteracted by MSAB (Figure 3A; Figure S3). At the protein level, β‐catenin, FZD8, and TCF7L2 were also notably elevated in RL‐QN15‐treated hESCs, but were substantially reduced by MSAB (Figure 3B). It is worth noting that MSAB inhibited the upregulation of both the 80 kDa (active, sumoylated) and 60 kDa (inactive, nonsumoylated) forms of TCF7L2. In addition, results showed that RL‐QN15 treatment significantly elevated mRNA levels of proliferation‐related genes *CCND1*, *MYC*, *PCNA*, and *MKI67*, which were effectively suppressed by MSAB (Figure 3A; Figure S3). At the protein level, RL‐QN15 increased the expression of CCND1, MYC, and PCNA, but this effect was reversed by MSAB treatment (Figure 3B). For migration‐related genes, RL‐QN15 treatment led to a significant elevation in mRNA levels of *MMP3*, *ITGA3*, *ITGA5*, and *ITGA6* in hESCs, which were notably reversed by MSAB (Figure 3A; Figure S3). Given that MMP3 is a secretory protein, we conducted an ELISA assay to measure its secretion level and found a notable increase in MMP3 secretion in RL‐QN15‐treated hESCs, which was mitigated by MSAB (Figure 3C). Furthermore, the pro‐stemness effect of RL‐QN15 on hESCs was significantly reduced by MSAB, as evidenced by the decrease in mRNA levels of *ITGB1*, *ITGA6*, and *LGR4* (Figure 3A; Figure S3). MSAB also decreased the protein levels of CK19, CK14, and LGR4, which were elevated in RL‐QN15‐treated hESCs (Figure 3B). During EMT, epithelial cells acquire mesenchymal characteristics, marked by a cadherin switch, which is noticeable by a decrease in CDH1 (also known as E‐cadherin) but an increase in CDH2 (also known as N‐cadherin). This transition is vital for morphogenesis and enhances cell migration and invasion, while the reduction of E‐cadherin correlates with the onset of EMT [53]. Our results indicated that RL‐QN15 treatment led to a significant decrease in CDH1 but an increase in CDH2, suggesting EMT enhancement to facilitate migration, which was significantly inhibited by MSAB (Figure 3A,B), corroborating the crucial role of Wnt/β‐catenin signaling in RL‐QN15‐induced EMT.

Overall, these results collectively demonstrated that RL‐QN15 promoted proliferation, migration, stemness, and the EMT process in hESCs through the β‐catenin signaling pathway.

### 2.3 RL‐QN15 Regulated hESCs’ Functions by Binding to FZD8

**FIGURE 4:**
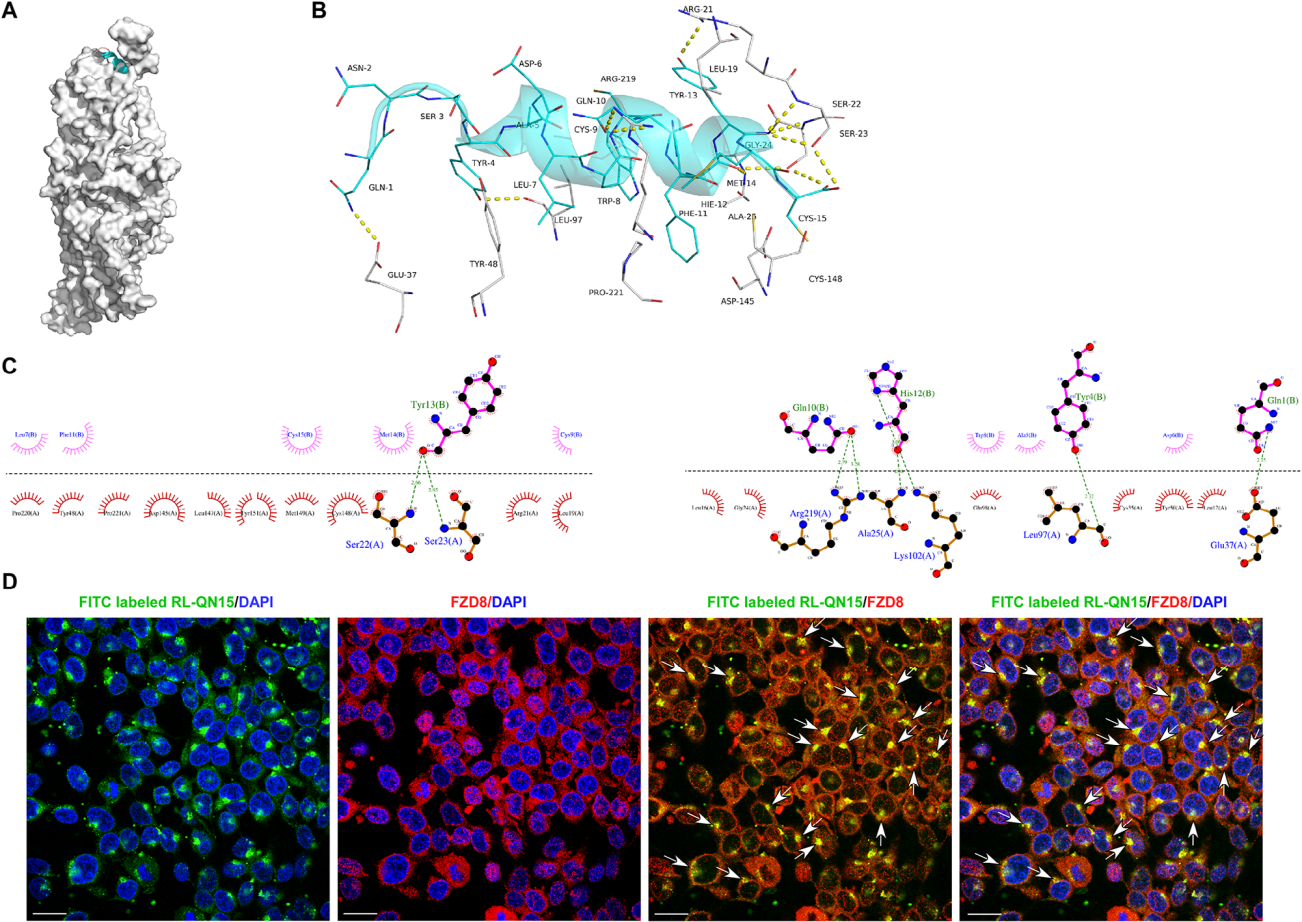
Analysis of molecular docking and co‐localization of RL‐QN15 and FZD8. (A) Representative image of RL‐QN15 docked with FZD8. (B) RL‐QN15 contained multiple interacting residues with FZD8, forming multiple hydrogen bonds (yellow dashed lines). (C) 2D interaction map of amino acid residues at different sites of RL‐QN15 with amino acid residues of FZD8. **(A)** represents FZD8, **(B)** represents RL‐QN15. (D) Representative images of immunofluorescence for co‐localization of FZD8 and RL‐QN15 in hESCs. FITC‐labeled RL‐QN15 (green), FZD8 (red), DAPI (blue). The white arrows indicated the representative binding sites of FZD8 and RL‐QN15. Scale bar: 20 µm.

Although we preliminarily concluded that RL‐QN15 stimulated the proliferation, migration, stemness, and EMT of hESCs by modulating the Wnt/β‐catenin signaling pathway, a deeper understanding of the underlying molecular mechanisms, particularly the specific cell membrane receptor interacting with RL‐QN15, remained elusive. The Wnt/β‐catenin signaling cascade encompasses numerous membrane receptors that mediate signal transmission. Among these, co‐receptors such as LRP5/6 amplify the signal, but none are as crucial as the frizzled receptor family. Frizzled (Fz) proteins serve as the primary receptors in the Wnt signaling pathway, distinguished by their seven‐transmembrane structure and an elongated N‐terminal cysteine‐rich domain [54]. As depicted in Figure 4A, molecular docking analysis suggested potential binding of RL‐QN15 to a groove within the extracellular region of the FZD8 receptor. This binding was mediated through electrostatic interactions, resulting in a compact complex formation. The binding free energy between RL‐QN15 and FZD8 was calculated to be −78 kcal mol^−1^, indicating an extraordinarily high binding affinity. Electrostatic interactions (−348 kcal mol^−1^) were pivotal in this interaction. Furthermore, multiple hydrogen bonds were identified between RL‐QN15 and FZD8, enhancing the stability of their association (Figure 4B,C; Figure S4A). Aromatic ring‐mediated interactions also contributed significantly to their binding efficiency (Figure S4B). To further validate the direct binding of RL‐QN15 to FZD8, a co‐localization experiment was conducted using immunofluorescence. The results revealed that green fluorescence‐labeled RL‐QN15 bound to red fluorescence‐labeled FZD8 on the cell membrane of hESCs, suggesting the direct binding of RL‐QN15 to the FZD8 receptor (Figure 4D).

**FIGURE 5:**
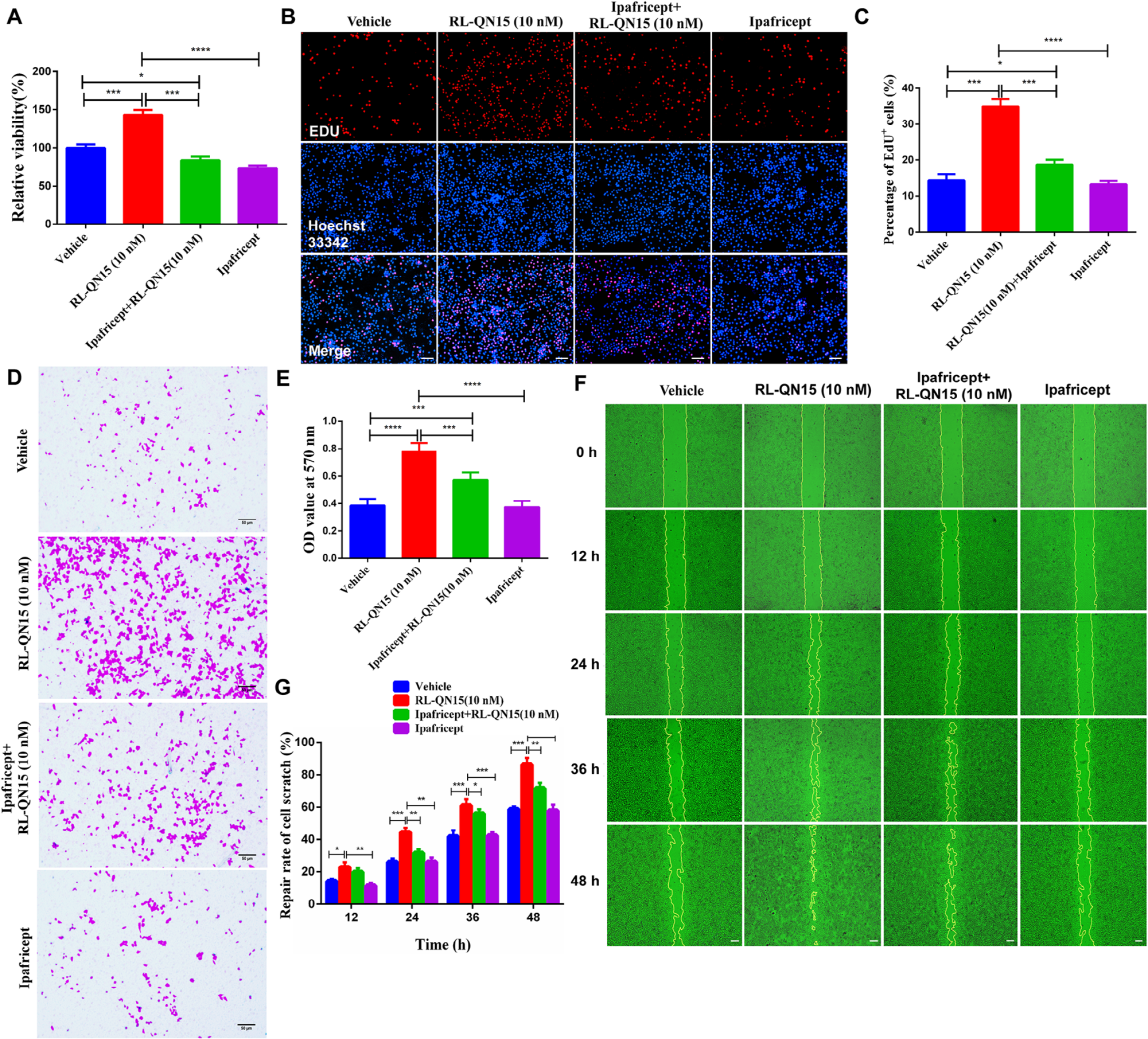
Effects of Ipafricept on proliferation, migration, and scratch repair ability of RL‐QN15‐treated hESCs. Quantitative assessment of the impact of Ipafricept on the RL‐QN15‐induced hESCs proliferation and viability (*n* = 5). (B,C) Representative photographs and quantitative analysis of EdU positive cells (red) in hESCs treated with Ipafricept in 10 nM RL‐QN15, respectively (*n* = 3). Scale bar: 50 µm. (D,E) Representative images and quantitative analysis of hESCs migration following RL‐QN15 treatment with or without Ipafricept (*n* = 5). Scale bar: 50 µm. (F,G) Representative images and quantitative analysis showing the effects of RL‐QN15 on hESCs scratch repair in the presence or absence of Ipafricept (*n* = 5). Scale bar: 100 µm. All data were expressed as mean ± SD, *p < 0.05, **p < 0.01, ***p < 0.001, ****p < 0.0001.

To further establish that RL‐QN15 activated the Wnt/β‐catenin signaling pathway through its binding to FZD8, thereby stimulating the proliferation, migration, stemness, and EMT of hESCs, we investigated whether Ipafricept, a decoy protein functioning as a specific antagonist for the FZD8 receptor [55], could mitigate or abolish the biological effects of RL‐QN15 on hESCs. The results of the MTS assay demonstrated that Ipafricept notably suppressed the proliferative effects of RL‐QN15 on hESCs (Figure 5A), which was further corroborated by EDU staining, showing a significant reduction in EDU‐positive cells in hESCs treated with both RL‐QN15 and Ipafricept compared to those treated alone with RL‐QN15 (Figure 5B,C). This underscored the inhibitory action of Ipafricept on RL‐QN15‐induced hESCs proliferation. The transwell assay exhibited a marked decrease in hESCs migration when cells were treated with both Ipafricept and RL‐QN15, as opposed to treatment with RL‐QN15 alone (Figure 5D,E). Additionally, the scratch assay results revealed that Ipafricept significantly hindered the scratch repair capabilities of RL‐QN15 on hESCs at various time intervals compared to treatment with RL‐QN15 alone (Figure 5F,G). These results collectively suggested that Ipafricept's inhibitory effects on hESCs proliferation and migration were pronounced in the presence of RL‐QN15, implying that the regulation of hESC proliferation and migration is orchestrated through the interaction between RL‐QN15 and FZD8.

**FIGURE 6:**
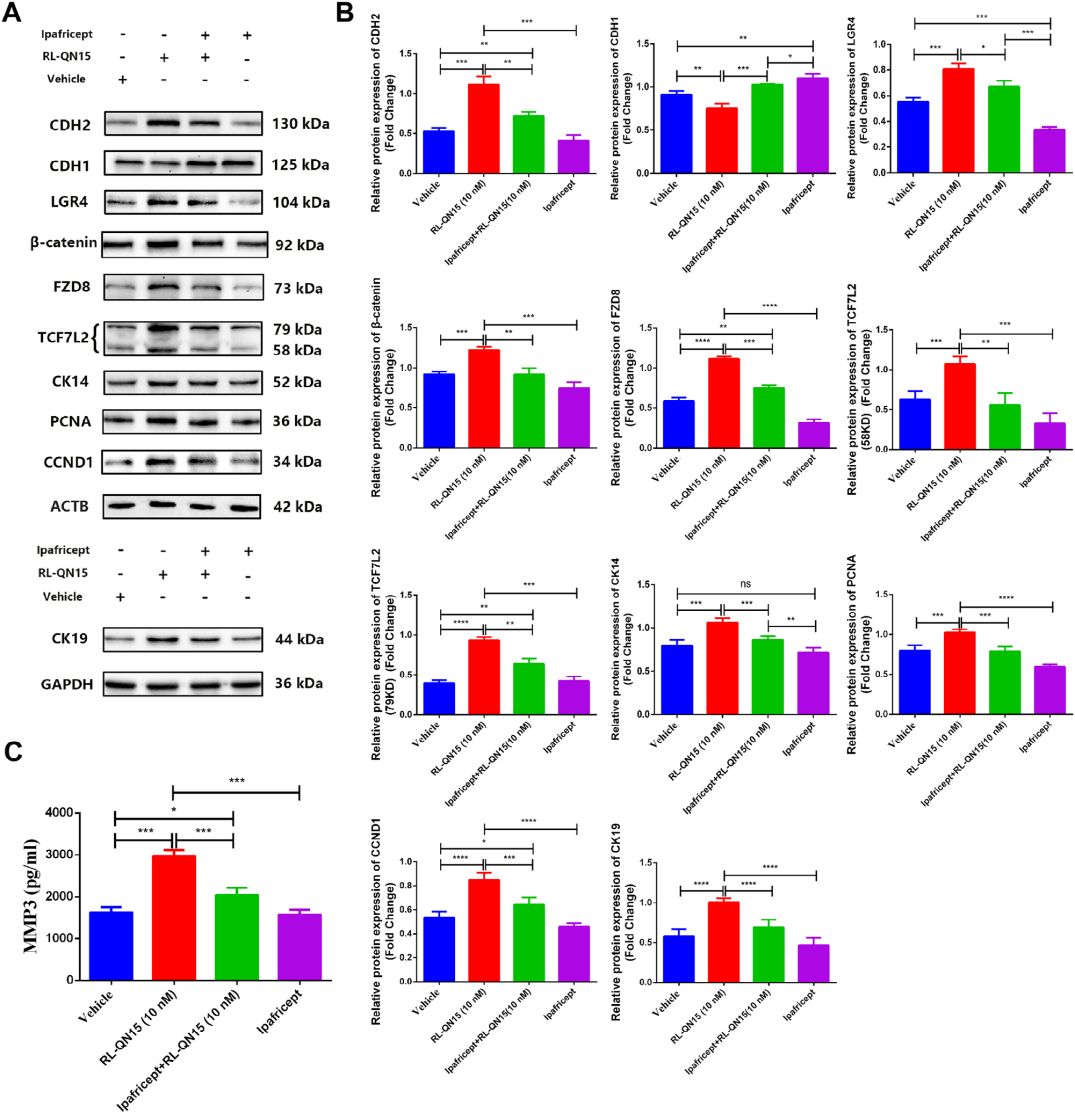
Effects of Ipafricept on Wnt/β‐catenin signaling pathway, proliferation, migration, stemness and EMT‐related protein expression in RL‐QN15‐treated hESCs. (A,B) Effects of Ipafricept on protein expression of key members of the Wnt/β‐catenin signaling pathway (β‐catenin, FZD8 and TCF7L2), proliferation‐related (CCND1 and PCNA), stemness‐related (CK19, CK14, and LGR4) and EMT‐related proteins (CDH1, CDH2) in 10 nM RL‐QN15‐treated hESCs. GAPDH and ACTB were used as internal references. (C) Effects of Ipafricept on MMP3 secretion in 10 nM RL‐QN15‐treated hESCs. All data were expressed as mean ± SD (*n* = 3), *p < 0.05, **p < 0.01, ***p < 0.001, ****p < 0.0001, ns, no significance.

To delve deeper into the mechanism by which RL‐QN15 modulates the Wnt/β‐catenin signaling pathway via its interaction with FZD8, thereby affecting hESCs function, we examined the influence of Ipafricept on the expression of pivotal Wnt/β‐catenin signaling pathway components and proteins associated with proliferation, migration, stemness, and EMT in hESCs treated with RL‐QN15. Our results revealed a substantial increase in the protein levels of key Wnt signaling pathway components, such as β‐catenin, FZD8, and TCF7L2, as well as proliferation‐related proteins CCND1 and PCNA, and the EMT marker protein CDH2, in hESCs treated with RL‐QN15. However, Ipafricept significantly inhibited this up‐regulation induced by RL‐QN15. Additionally, Ipafricept effectively reversed the RL‐QN15‐mediated down‐regulation of CDH1 (Figure 6A,B). Furthermore, Ipafricept notably reduced the stemness enhanced by RL‐QN15, as evidenced by a considerable decrease in levels of CK19, LGR4, and CK14 (Figure 6A,B). Using ELISA, we detected significantly elevated levels of MMP3 secretion in hESCs treated with RL‐QN15, which were effectively reversed by Ipafricept administration (Figure 6C). These findings collectively suggested that Ipafricept disrupted the interaction between RL‐QN15 and FZD8 by competing for binding, leading to a marked reduction in the binding of RL‐QN15 to the FZD8 receptor on the cell membrane surface. Consequently, Ipafricept reversed the activation of the Wnt/β‐catenin signaling pathway induced by RL‐QN15, thereby reversing the effects of RL‐QN15 on the proliferation, migration, stemness, and EMT of hESCs.

### 2.4 RL‐QN15 Accelerated Wound Healing by Binding With FZD8 and Regulating ESCs Functions

**FIGURE 7:**
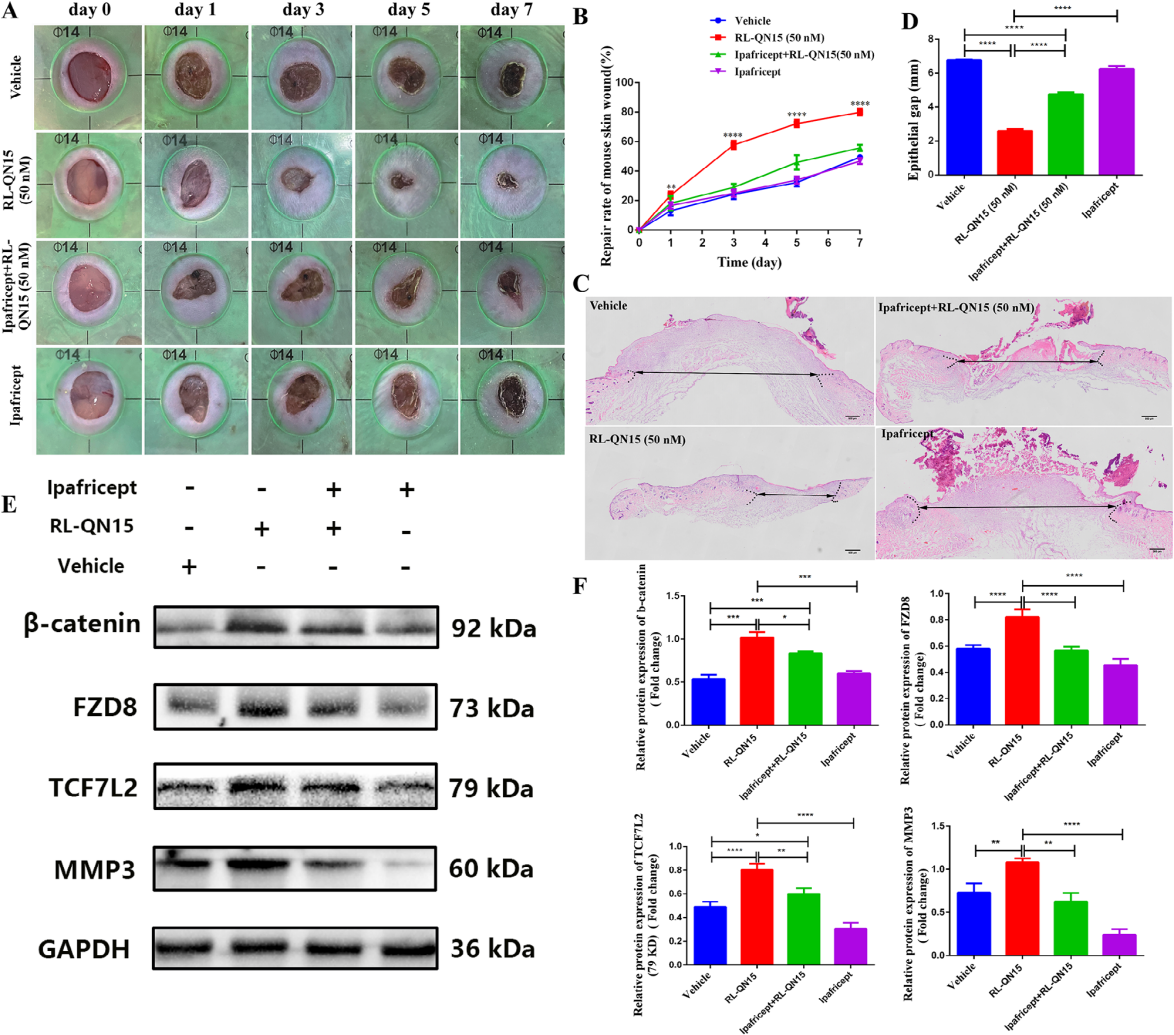
Effect of Ipafricept on wound healing ability and activation of Wnt/β‐catenin signaling pathway mediated by RL‐QN15 in mice. (A) Representative image of healing progress in four groups of mice from days 0 to 7. (B) Statistical analysis of wound healing rate in four groups of mice on days 1, 3, 5, and 7 (*n =* 5 per group). Wound closure rate was calculated as % of wound closure = 100 × (initial wound area − unhealed wound area) / initial wound area. (C) H&E staining of mouse tissues on postoperative day 7 to detect mature epidermal gap in each group. Mature epidermal boundary was outlined with a black dotted line, and length of arrow between dotted lines represented width of mature epidermal gap. Scale bar: 500 µm. (D) Statistical analysis of mature epidermal gap in different groups (*n =* 5 per group). (E,F) Effects of Ipafricept on protein expression of key members of the Wnt/β‐catenin signaling pathway (β‐catenin, FZD8, and TCF7L2) and migration‐related proteins (MMP3) in 50 nM RL‐QN15‐treated wound skin of mice. GAPDH was used as internal references. (*n* = 3). All data were expressed as mean ± SD, *p < 0.05, **p < 0.01, ***p < 0.001, ****p < 0.0001.

The aforementioned in vitro studies suggested that RL‐QN15 augments stemness, proliferation, migration, and EMT in ESCs by engaging with the FZD8 membrane receptor, thereby activating the down‐stream Wnt/β‐catenin signaling pathway. To evaluate the in vivo impact of RL‐QN15's binding to FZD8 on ESCs and to further verify that RL‐QN15 enhanced wound healing by modulating ESCs function, we next assessed its regenerative potential in mouse full‐thickness skin injury model. After 7 days, the skin wound healing rate in the RL‐QN15 group neared 90%, whereas the rate in the Ipafricept + RL‐QN15 group was less than 60% (Figure 7A,B). Successful wound healing hinges on proliferative epithelization, without it, skin wound recovery cannot be achieved [54]. Both ESCs and their differentiated descendants play crucial roles in re‐epithelialization [56, 57]. Notably, compared to the vehicle group, the RL‐QN15 group exhibited a reduced epidermal gap on postoperative day 7, as observed through H&E staining. This gap was significantly wider in the Ipafricept + RL‐QN15 group compared to the RL‐QN15 group (Figure 7C,D), indicating that Ipafricept substantially inhibited RL‐QN15‐induced re‐epithelialization. Furthermore, on postoperative day 7, the RL‐QN15 group had a thinner neo‐epidermal layer compared to the vehicle group, while the Ipafricept + RL‐QN15 group had a thicker layer compared to the RL‐QN15 group (Figure S5A,B). These findings suggested that RL‐QN15 might facilitate the healing of full‐thickness cortical wounds through interactions with FZD8, while Ipafricept significantly impeded this process. To clarify the role of Wnt/β‐catenin signaling pathway activation in wound healing following the binding of RL‐QN15 to FZD8, skin tissue specimens were collected from mouse wounds on postoperative day 7 for western blotting of key signaling components. The analysis revealed that the protein levels of β‐catenin, FZD8, and TCF7L2 were notably higher in the RL‐QN15‐treated group than in the vehicle group but were substantially suppressed by Ipafricept treatment. Our findings indicated that TCF7L2 primarily exists in its activated form (79 kDa) within skin wound tissue (Figure 7E,F). Additionally, the migration‐related protein MMP3 was significantly elevated after RL‐QN15 treatment but was markedly reduced after Ipafricept administration (Figure 7E,F). These results implied that Ipafricept competes with FZD8 for binding to RL‐QN15, thereby inhibiting their interaction and counteracting the activation of the Wnt/β‐catenin signaling pathway by RL‐QN15.

**FIGURE 8:**
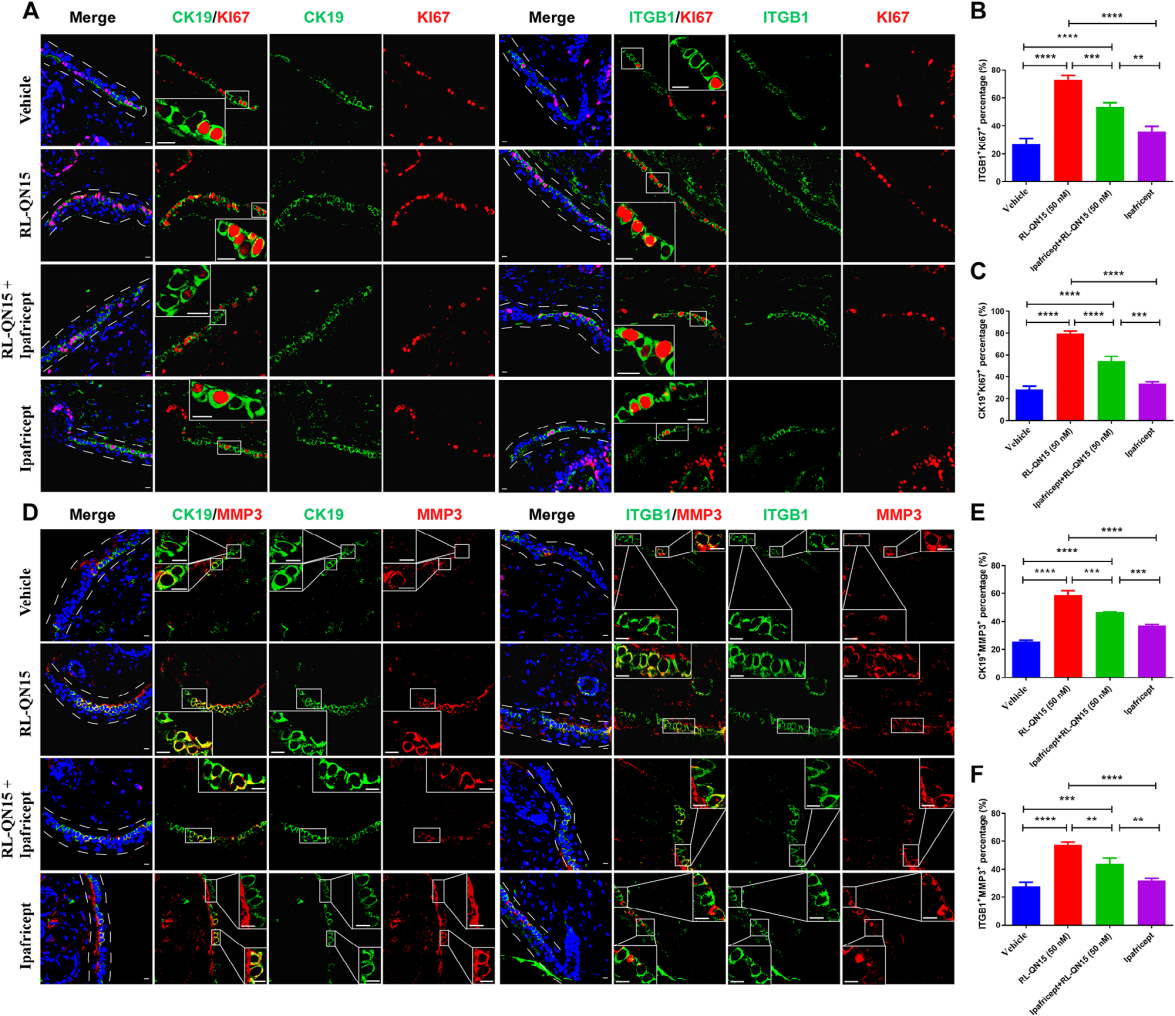
The effect of RL‐QN15 and Ipafricept on ESCs proliferation and migration in mouse skin wounds. (A) Representative image of effects of Ipafricept and RL‐QN15 on protein expression of CK19, ITGB1, and KI67 in mouse skin wounds by immunofluorescence staining of frozen sections. Insets showed the higher magnification of representative area indicated by squares. Scale bar: 10 µm. (B,C) Percentage of proliferating ESCs in skin wound tissue from four groups of mice, with five fields per mouse randomly selected to calculate percentage of CK19^+^KI67^+^/CK19^+^and ITGB1^+^KI67^+^/ITGB1^+^ cells. (D) Representative images of effects of Ipafricept and RL‐QN15 on protein expression of CK19, ITGB1, and MMP3 in mouse skin wounds by immunofluorescence staining of frozen sections. Insets showed the higher magnification of representative area indicated by squares. Scale bar: 10 µm. (E,F) Percentage of migrating ESCs in skin wound tissue from four groups of mice, with five fields per mouse randomly selected to calculate percentage of CK19^+^MMP3^+^/CK19^+^ and ITGB1^+^MMP3^+^/ITGB1^+^ cells. All data were expressed as mean ± SD (*n* = 3), **p < 0.01, ***p < 0.001, ****p < 0.0001.

To ascertain whether RL‐QN15‐mediated activation of the Wnt/β‐catenin pathway in mice augmented the proliferation, migration, and stemness of ESCs, skin tissue was harvested from mouse wounds on postoperative day 7 and subjected to immunofluorescence staining of frozen sections. We then evaluated the abundance of proliferating and migrating ESCs in the wounded skin and our results indicated that the RL‐QN15 group exhibited a substantial increase in the proportion of CK19^+^KI67^+^ and ITGB1^+^KI67^+^ cells compared to the vehicle group, with percentages rising from approximately 30% to roughly 80%. This suggested that RL‐QN15 promoted ESCs proliferation in wounded skin. Conversely, the combination of Ipafricept and RL‐QN15 led to a notable decrease in the proportion of these cells compared to the RL‐QN15 group, with percentages dropping from 80% to 55% (Figure 8A–C). Furthermore, compared to the vehicle group, the RL‐QN15 group demonstrated a significant rise in CK19^+^MMP3^+^ and ITGB1^+^MMP3^+^ cells, with percentages increasing from 25% to 60%. This suggested that RL‐QN15 significantly enhanced ESCs migration in skin wounds. These findings were corroborated by western blotting results showing increased MMP3 protein expression in tissues (Figures 7E,F and 8D–F). However, the combination of Ipafricept and RL‐QN15 resulted in a notable reduction in the proportion of CK19^+^MMP3^+^ and ITGB1^+^MMP3^+^ cells compared to the RL‐QN15 group, with percentages decreasing from approximately 60% to around 45% (Figure 8D–F). Concurrently, the count of ESCs (CK19^+^/ITGB1^+^ cells) in five microscopic fields per mouse was notably higher in the RL‐QN15 group compared to the vehicle group, but decreased after Ipafricept intervention (Figure 8A,D; Figure S5C,D). In conclusion, RL‐QN15 activated the Wnt/β‐catenin signaling pathway during skin wound healing in mice by binding to the membrane receptor FZD8, thereby enhancing ESCs proliferation, migration, and stemness, and ultimately facilitating wound healing.

## 3 Discussion

Currently, small molecule compounds and growth factors dominate pharmaceutics used in wound healing. Nevertheless, the inherent limitations have hampered their application [58]. Hence, skin wound repair remains challenging, the existing medications struggle to meet rising clinical demands. Peptide drugs have gained prominence due to their significant advantages [21, 59, 63]. Our research group has previously identified several wound healing peptides from amphibians [18, 64, 65], a renowned source of active peptides, such as RL‐RL10, OA‐GP11 [66], OA‐GL17d [67], and RL‐QN15 [10]. Among them, RL‐QN15 shows remarkable efficacy in healing acute and chronic wounds, skin fibrosis, and oral ulcers, possessing great potential as a new type of wound healing promoting drug. However, the mechanism of RL‐QN15 in promoting wound healing remains poorly understood, impeding its clinical candidacy. ESCs are the most crucial functional cells for skin tissue damage repair. Regulating the functions of ESCs, particularly proliferation and migration, notably enhance wound healing [25, 26, 30, 31, 68, 69]. The rapid proliferation and migration of ESCs are vital for wound closure and the restoration of normal skin function. Hence, this study aims to investigate the novel mechanism of amphibian‐derived peptide RL‐QN15 in promoting wound healing from the fresh perspective of ESCs, filling the gap in the regulation of ESCs function by amphibian‐derived peptides.

Our in vitro experiments demonstrated that RL‐QN15 notably enhanced hESCs proliferation, migration, stemness, and EMT by activating the Wnt/β‐catenin signaling pathway. The Wnt signaling pathway in wound tissue is intricately associated with cell proliferation, migration, polarity, and the maintenance of stemness [70]. Modification of the Wnt/β‐catenin signaling pathway is pivotal in the molecular mechanism underlying atypical wound healing and scar formation. We innovatively explored the role of the Wnt/β‐catenin signaling pathway in wound repair through the specific regulation of ESCs. As a key member of the frizzled protein family, the FZD8 receptor plays a significant role in the Wnt/β‐catenin signaling pathway. Although previous studies have found that elevated expression of FZD8 can promote the activation of Wnt/β‐catenin signaling pathway [71, 72], the effect of FZD8 on ESCs function remains unexplored. Our results revealed that RL‐QN15 interacted with FZD8 receptor located in the membranes of hESCs via electrostatic interactions to form a stable complex and further activate Wnt/β‐catenin signaling pathway. However, Ipafricept, competing with FZD8 for RL‐QN15 binding, inhibited the activation of RL‐QN15‐mediated Wnt/β‐catenin signaling pathway and further disrupted the biological processes of proliferation, migration, stemness, and EMT of hESCs. These results regarding the involvement of FZD8 in regulating hESCs function represent a novel discovery in the field.

As a key member of the MMP family, the MMP3 promoter contains TCF binding sites, rendering it a potential target for β‐catenin/TCF interaction [73]. Our findings indicated that the activation of the Wnt/β‐catenin pathway induced by RL‐QN15 was accompanied by a significant up‐regulation in MMP3 expression. Studies have demonstrated that the non‐catalytic HPX domain of MMP3 binds to and inactivates the non‐canonical Wnt ligand Wnt5b, thereby promoting activation of the Wnt/β‐catenin signaling pathway in breast stem cells [74], implying that elevated expression of MMP3 may further promote Wnt/β‐catenin signaling pathway activation in hESCs. CDH1 (E‐cadherin) can be hydrolyzed by MMP3, thereby reducing cell adhesion and promoting cell migration [75]. In hESCs treated with RL‐QN15, a significant decline in CDH1 (E‐cadherin) expression and a considerable increase in CDH2 (N‐cadherin) expression were observed, indicating the occurrence of EMT, which is a crucial part in wound healing. The activation of the Wnt/β‐catenin signaling pathway mediated by RL‐QN15 and its binding to FZD8 are instrumental in initiating EMT. Cadherins are transmembrane adhesion molecules, and their cytoplasmic segments bind to various molecules, including p120‐catenin, β‐catenin, and α‐catenin, forming the cadherin‐catenin complex [76]. This complex plays a pivotal role in cell adhesion and interaction and regulates the Wnt/β‐catenin signaling pathway by sequestering β‐catenin to the cell membrane [77]. The reduction in E‐cadherin expression not only leads to the separation of β‐catenin from the complex and its nuclear translocation to initiate LEF/TCF‐mediated transcription and activate Wnt/β‐catenin signaling pathway further, but also triggers an increase in N‐cadherin expression and the initiation of the EMT process [76, 78]. Increased N‐cadherin expression results in the accumulation of β‐catenin on the cell membrane, thereby reducing the nuclear Wnt/β‐catenin signaling [79], potentially serving as one of the mechanisms to restrict RL‐QN15‐induced Wnt signaling pathway overactivation and ESCs functions overregulation, ensuring normal skin structure and less scarring during RL‐QN15‐mediated wound healing. Previous studies have shown that EMT is activated during wound healing, and Wnt/β‐catenin signaling pathway can trigger EMT [80], furthermore, FZD8 is linked to the expression of genes associated with EMT [81], which are highly consistent with our findings.

Based on the aforementioned in vitro experimental findings, we confirmed the interaction between RL‐QN15 and FZD8 and effects of their binding on ESCs function and wound healing by utilizing full‐thickness skin wound model in mice. The results indicated that the ability of RL‐QN15 to promote wound epithelialization and healing is impaired by Ipafricept. Meanwhile, consistent with in vivo findings, the improvements in the proliferation, migration, and stemness of ESCs mediated by RL‐QN15 were also reversed by Ipafricept. These data indicate that RL‐QN15, both in vitro and in vivo, promotes Wnt/β‐catenin signaling pathway activation by binding with FZD8 and enhances the proliferation, migration, and stemness of ESCs further, thereby expediting wound healing. This study is the first report to demonstrate that FZD8 is directly involved in wound healing.

In summary, our study demonstrated that RL‐QN15 activates the Wnt/β‐catenin signaling pathway by binding to FZD8 membrane receptor both in vitro and in vivo. This activation elevates proliferation‐related target genes expression, such as MYC and CCND1, facilitating ESCs replication. The enhanced expression of target gene *MMP3* promotes ESCs migration and activates Wnt/β‐catenin signaling pathway further. Notably, MMP3 mediated E‐cadherin hydrolysis allows β‐catenin nuclear entry, activating Wnt/β‐catenin signaling pathway and further stimulating EMT and promoting the proliferation, migration, and stemness of ESCs in turn. Through the above mechanisms, RL‐QN15 achieves the efficacy of facilitating wound healing. These findings uncover a novel mechanism by which amphibian‐derived peptides accelerate skin wound healing via ESC regulation, identifying FZD8 as a new target for skin trauma treatment. In addition, unlike Wnt proteins with limited expression, solubility, and in vivo pharmacokinetics [82], RL‐QN15 may serve as a natural FZD8‐selective Wnt surrogate that binds to FZD8 to activate the Wnt/β‐catenin signaling pathway and holds broad clinical application prospects.

Despite these findings, several issues remain unresolved in our study. For instance, the precise mechanism by which RL‐QN15 elevates FZD8 expression remains unclear. Related studies will more comprehensively reveal the regulatory mechanism of RL‐QN15 on the function of ESCs and enrich our understanding of amphibian‐derived peptides. ESCs treated with RL‐QN15 combined with MSAB/Ipafricept exhibited enhanced proliferation, migration, stemness, and EMT compared to the vehicle group. Similarly, wounds treated with RL‐QN15 combined with Ipafricept healed faster than those in the vehicle group, suggesting that RL‐QN15 may activate additional signaling pathways beyond the Wnt/β‐catenin signaling pathway. The potential interplay and cooperation between these pathways in ESCs regulation and wound healing warrant further exploration. In addition, ESCs hold significant prospects in skin trauma treatment. At present, autologous transplantation is the preferred clinical treatment for skin injury [83]. However, the low ESCs content often leads to clinical implantation of autologous epidermal grafts (CEA) failure. Enhancing ESCs could improve skin substitutes and CEA transplantation efficacy [84, 85, 86, 87]. Given that RL‐QN15 has been shown to promote ESCs proliferation and migration, it may reduce the CEA production time. Future research should explore the combination of RL‐QN15 and biomaterial scaffolds to accelerate the tissue‐engineered skin development.

## 4 Methods

### 4.1 Cell Culture and Phenotype Identification

The hESCs were cultured following previously published studies [27, 44, 88], with some adjustments. Briefly, the cells were cultivated in serum‐free EpiLife medium (Gibco, USA) supplemented with HKGS (Thermo, USA). The culture flasks were coated with collagen IV (Sigma, USA). Flow cytometry was used to identify cell identity. Briefly, after fixation, permeabilization, and blocking, cells were incubated with CK19 and ITGβ1 for 30 min, followed by secondary antibodies for 30 min. Fluorescent signal detection was performed using flow cytometry. Primary antibodies included CK19 (Abcam, UK) and ITGβ1 (Abcam, UK) and fluorescent secondary antibodies included CoraLite488 conjugate goat anti‐mouse IgG (H+L) (Proteintech, USA) and Cy3‐conjugated Affinipure goat anti‐rabbit IgG (H+L) (Proteintech, USA). Positive cell percentages were analyzed using FlowJo software.

### 4.2 Cell Proliferation

The effect of RL‐QN15 (Purity > 98%, Wuhan Tanda Biotechnology Co., LTD) on hESCs proliferation was assessed using MTS assay and EDU staining. For MTS assay, the hESCs were seeded in collagen IV‐coated 96‐well plates and cultured with or without MSAB (methyl 3‐(4‐methylphenylsulfonamido)benzoate) (10 µM, MedChemExpress, USA) and Ipafricept (10 µg mL^−1^, MedChemExpress, USA) in different concentrations of RL‐QN15. As a vehicle group, the same amount of EpiLife medium was added without any other substances. After 1, 2, 3, and 4 days, 20 µL MTS solution (Promega, USA) in CellTiter 96AQueous One Solution kit was introduced into each well. After 4 h of incubation, the absorbance value of each sample in the groups was then measured using a microplate reader (Spectra Max 190, USA) at 490 nm.

For EDU staining, the hESCs were seeded on collagen IV‐coated cell climbing sheets and cultured with or without MSAB (10 µM, MedChemExpress, USA) and Ipafricept (10 µg mL^−1^, MedChemExpress, USA) in RL‐QN15 (10 nM). After incubation with EDU (Beyotime, China) at 10 µM for 2 h, the cells were fixed and permeabilized. Subsequently, 0.5 mL of Click working solution was introduced into each well, followed by 30 min of incubation. Hoechst 33342 dye was employed for the visualization of cell nuclei. The slides were then mounted using 80% glycerol. Fluorescence microscopy was employed to observe the positive cells. ImageJ software was utilized to assess the proportion of actively dividing cells.

### 4.3 Cell Cycle

The cell cycle was determined by DNA content quantification analysis. The hESCs were cultured in 6‐well plates coated with collagen IV and randomly set as the vehicle group and RL‐QN15 (10 nM) group. After 56 h and 3 days of cultivation, the cells were collected and fixed in cold anhydrous ethanol overnight at 4°C. Flow cytometry was employed to identify fluorescence signals after incubation with propidium iodide staining solution in a light‐restricted environment for 30 min. FlowJo software was utilized for evaluating the cellular distribution at each phase.

### 4.4 Cell Scratch Assay

The impact of RL‐QN15 on the scratch healing capability of hESCs was evaluated using a cell scratch assay. hESCs were seeded in 24‐well plates coated with collagen IV, and incubated with EpiLife complete medium containing 1% HKGS for 12 h. Subsequently, hESCs were transitioned to EpiLife medium devoid of any additives and cultured until reaching approximately 90% confluence. To inhibit cell proliferation, 8 µg mL^−1^ mitomycin (Sigma, USA) was administered at 37°C for 4 h. The sterile pipette tip (Axygen, USA) was utilized to scrape the cells, creating mechanical wounds. Cells were cultured with or without MSAB (10 µM, MedChemExpress, USA) and Ipafricept (10 µg mL^−1^, MedChemExpress, USA) in different concentrations of RL‐QN15. The photographs were captured at 0, 12, 24, 36, and 48 h to assess the scratch repair rate.

### 4.5 Cell Migration Assay

The migration chambers equipped with a polycarbonate membrane having a pore size of 8 µm (Corning, USA) were used to assess the influence of RL‐QN15 on the migratory ability of hESCs. Cells were suspended in EpiLife medium (without HKGS) and 100 µL cell suspension was added into the upper chamber of collagen‐coated transwell 24‐well plates. Then 600 µL medium containing RL‐QN15 (10 nM) was added in the lower chamber with or without MSAB (10 µM, MedChemExpress, USA) and Ipafricept (10 µg mL^−1^, MedChemExpress, USA). After culturing for 36 h, the cells were stained with a crystal violet staining reagent at a concentration of 0.1% for 20 min, and then wiped off the filter membrane surface. Cell migration was examined under microscope (Leica DM4B, Germany), and five fields of view were randomly chosen and analyzed.

### 4.6 Quantitative Real‐Time Polymerase Chain Reaction (qRT‐PCR)

qRT‐PCR was used to assess gene expression. Briefly, hESCs were seeded into collagen‐coated 6‐well plates and cultured with or without MSAB (10 µM, MedChemExpress, USA) in 10 nM RL‐QN15. After 3 days, the total RNA extraction kit (TIANGEN, China) was employed for the isolation of total RNA from hESCs in each group. The mRNA reverse transcription was then carried out with the SureScript First‐Strand cDNA Synthesis Kit (GeneCopoeiaTM, USA) and qPCR analysis was conducted by using BlazeTaq SYBR Green qPCR mix2.0 (GeneCopoeiaTM, USA). The primer sequences are exhibited in Table S1. GAPDH was used as an internal reference, and the 2^−△△^ *^CT^* method was utilized to ascertain relative expression.

### 4.7 Western Blotting

The efficient RIPA lysate (Solarbio, China) was combined with PMSF (Solarbio, China) at a 100:1 ratio to extract total protein from cells or mouse wound tissues. The Bradford method (BCA protein analysis kit, Solarbio, China) was utilized to quantify the extracted proteins. Subsequently, the proteins were mixed with 4× loading buffer at a 1:4 volume ratio and denatured at 95°C for 15 min. Equal amounts of proteins (20–30 µg) were then separated by 10% SDS‐PAGE gel and transferred onto 0.45 µm PVDF membrane via a semi‐dry method. Blots were treated with 5% non‐fat dried milk for 2 h at room temperature, then incubated with primary antibodies at 4°C overnight (for at least 16 h), and with secondary antibodies for 1 h at room temperature. The primary antibodies included: anti‐active β‐catenin (Cell Signaling, USA), anti‐CDH2 (Proteintech, USA), anti‐TCF7L2 (Cell Signaling, USA), anti‐MMP3 (Proteintech, USA), anti‐FZD8 (Proteintech, USA), anti‐CCND1 (Proteintech, USA), anti‐MYC (Proteintech, USA), anti‐PCNA (Proteintech, USA), anti‐CDH1 (Proteintech, USA), anti‐LGR4 (Proteintech, USA), anti‐CK14 (Proteintech, USA), anti‐GAPDH (Proteintech, USA), and anti‐ACTB (Proteintech, USA). The secondary antibodies consisted of HRP‐conjugated Affinipure Goat Anti‐Rabbit IgG(H+L) (Proteintech, USA) and HRP‐conjugated Affinipure Goat Anti‐Mouse IgG (H+L) (Proteintech, USA). The ECL kit (Biosharp, China) was responsible for the formation of bands, which were then exposed using the Gel imaging instrument (BIO‐RAD, Chemidoc XRS+, USA). The Image Lab software (BIO‐RAD, USA) was employed to analyze the protein bands, and quantitative analysis was conducted using GraphPad Prism 7.

### 4.8 Molecular Docking

The interaction between RL‐QN15 and FZD8 was explored via molecular docking. Briefly, using the i‐Tasser online server (https://zhanggroup.org/I‐TASSER), the three‐dimensional (3D) structure of FZD8 was constructed. To simulate the structure of peptide ligands, the PEP‐FOLD3 online server (https://bioserv.rpbs.univ‐paris‐diderot.fr/services/PEP‐FOLD3/) was employed, generating RL‐QN15 for subsequent protein‐peptide docking using the Z‐dock server (https://zdock.umassmed. edu/). The conformation with the highest score was chosen, amber18 software package was used to simulate the molecular dynamics of the protein‐peptide complex. Hydrogen atoms and antagonistic ions were added to neutralize the charge. The MMPBSA.py module was used to ascertain the binding free energy between the peptide and protein.

### 4.9 Co‐Localization of FZD8 and RL‐QN15

The hESCs were seeded on collagen IV‐coated cell‐climbing sheets. After attachment, cells were treated with FITC‐labeled RL‐QN15 for 1 h, followed by PBS washing and fixation with 4% PFA. After blocking with 5% BSA, hESCs were incubated with FZD8 primary antibody (Proteintech, USA) for 16 h at 4°C, and then incubated with Cy3‐conjugated affinipure goat anti‐rabbit IgG (H+L) (Proteintech, USA) for 1 h. After DAPI staining and sealing, the co‐localization of FZD8 and RL‐QN15 was observed and analyzed by a laser confocal microscope (SpinSR, Olympus, Japan).

### 4.10 Animal Ethics Statement

All animal care and experiments were performed in accordance with the ARRIVE guidelines. Male Kunming mice (28–30 g, 6–8 weeks old) were purchased from Hunan SJA Laboratory Animal Co. (Hunan, China). All animal care and experiments were approved by the Kunming Medical University Ethics Committee (approval No.: Kmmu20240247).

### 4.11 Establishment and Application of Full‐Thickness Skin Wound Model in Mice

Male Kunming mice (Mus musculus, Kunming strain), weighing 28–30 g and aged 6–8 weeks, were purchased from Hunan SJA Laboratory Animal Co. (Hunan, China). The mice were housed in individually ventilated cages (FENGSHI, China) at the animal facility of Kunming Medical University, given ad libitum access to food and water, and maintained under a 12‐h light/dark cycle. A full‐thickness injured skin wound model was established in mice to evaluate the therapeutic efficacy of RL‐QN15 (50 nM) and the influence of Ipafricept on wound healing [10]. After 1 week of acclimation, the mice were anesthetized by intraperitoneal injection of 1% sodium pentobarbital (0.1 mL/20 g body weight). The dorsal hair of the mouse was shaved, and the skin was disinfected with a 70% ethanol swab to prepare for the creation of a standardized full‐thickness cutaneous wound. Two 10 mm×10 mm full‐thickness excisional wounds were created on the back of each mouse. At the end of the surgical procedure, cages were moved close to a heating apparatus until the mice fully recovered from anesthesia.

Once the wound model was established, mice were randomly divided into four groups, each comprising five mice: the Vehicle group (PBS), the RL‐QN15 group (50 nM), the Ipafricept (10 mg kg^−1^) + RL‐QN15 (50 nM) group, and the Ipafricept (10 mg kg^−1^) group. RL‐QN15 was administered to the wound locally twice daily. Subcutaneous injections of 10 mg kg^−1^ Ipafricept were administered at multiple points around the wound on days 0 and 3. The progression of wound healing was monitored by photographing the wound area every other day.

### 4.12 Histological Analysis

On postoperative day 7, mice were euthanized, and the tissue around the wound, including 2 mm of normal skin, was collected. After fixation and dehydration, tissues were embedded with OCT and followed by frozen section. Sections were stained by HE, and epidermal gap and thickness were calculated for each group. ImageJ and GraphPad Prism were used to analyze and quantify the images.

### 4.13 Tissue Immunofluorescence Staining

After antigen retrieval, permeabilizing and blocking, the frozen sections of the specimen were incubated with primary antibody at 4°C for 16 h, then incubated with secondary antibody for 1 h at room temperature. Then, the sections were rinsed and subsequently incubated with the second primary antibody and secondary antibody sequentially. Nuclei were labeled by DAPI and mounting coverslips with 80% glycerol. Olympus fv1000 laser confocal microscope was used to capture the images, and FV10‐ASW 2.1 software was used to edit and analyze images. Primary antibodies included: anti‐CK19 (Proteintech, USA), anti‐ITGB1 (Santa Cruz, USA), anti‐KI67 (Abcam, UK), and anti‐MMP3 (Abcam, UK). The secondary antibodies included CoraLite488 conjugate Goat Anti‐Mouse IgG(H+L) (Proteintech, USA) and Cy3‐conjugated Affinipure Goat Anti‐Rabbit IgG(H+L) (Proteintech, USA).

### 4.14 Enzyme‐Linked Immunosorbent Assay (ELISA)

The hESCs were seeded in collagen IV‐coated 24‐well plates and cultured with or without MSAB (10 µM, MedChemExpress, USA) and Ipafricept (10 µg mL^−1^, MedChemExpress, USA) in RL‐QN15 (10 nM) until about 90% confluence. The cell supernatants of each group were collected and centrifuged at 1 000 ×*g* for 20 min at 4°C. The MMP3 expression levels of each group were determined using the Human MMP3 ELISA kit (NeoBioscience, China.).

### 4.15 Statistical Analysis

Statistical analysis was performed using GraphPad Prism 7.0 software, and all data were expressed as mean ± standard deviation. For comparison of two groups, Student's *t*‐test was used. One‐way ANOVA followed by Tukey's post hoc test was used to compare multiple groups. Two‐way ANOVA test was performed to assess the influence of two factors on the dependent variable. *P* < 0.05 was considered statistically significant, and all experiments were performed in triplicate at least.

## Author Contributions

Xinwang Yang designed and supervised the project. Xinwang Yang, Ying Wang, Li He, and Kun Guo received financial support for this project. Xinwang Yang and Jun Sun provided feedback regarding the project. Yuansheng Li and Qiuye Jia participated in all experimental investigations and data analysis. Naixin Liu, Saige Yin, Junyuan Wang, and Shaoyang Zhang conducted experiments in vivo and examined the resulting data. Yuliu Yang, Ying Peng, Bu'er Qi, and Zeqiong Ru performed in vitro experiments and analyzed the data. Yuansheng Li and Kun Guo, Ying Wang, and Xinwang Yang wrote and revised the paper. All authors actively participated in the project's discussions. The final manuscript was reviewed and endorsed by all authors.

## Conflicts of Interest

The authors declare no conflicts of interest.

## Supporting information




**Supporting File**: exp270057‐sup‐0001‐SuppMat.pdf

## Data Availability

The datasets used and/or analyzed in the current study will be provided by the corresponding author upon reasonable request.

## References

[1] Z. Meng , D. Zhou , Y. Gao , M. Zeng , and W. Wang , “miRNA Delivery for Skin Wound Healing,” Advanced Drug Delivery Reviews 129 (2018): 308–318. 29273517 10.1016/j.addr.2017.12.011

[2] J. Mwangi , P. Kamau , R. Thuku , and R. Lai , “Design Methods for Antimicrobial Peptides With Improved Performance,” Zoological Research 44 (2023): 1095. 37914524 10.24272/j.issn.2095-8137.2023.246 PMC10802102

[3] S. Yin , Y. Wang , and X. Yang , “Amphibian‐Derived Wound Healing Peptides: Chemical Molecular Treasure Trove for Skin Wound Treatment,” Frontiers in Pharmacology 14 (2023): 1120228. 37377928 10.3389/fphar.2023.1120228 PMC10291078

[4] H. Lu , J. Chai , Z. Xu , et al., “Cath‐KP, A Novel Peptide Derived From Frog Skin, Prevents Oxidative Stress Damage in a Parkinson's Disease Model,” Zoological Research 45 (2024): 108–124. 38114437 10.24272/j.issn.2095-8137.2023.101 PMC10839659

[5] X. Li , Y. Wang , Z. Zou , et al., “OM‐LV20, A Novel Peptide From Odorous Frog Skin, Accelerates Wound Healing In Vitro and In Vivo,” Chemical Biology & Drug Design 91 (2017): 126–136. 28650592 10.1111/cbdd.13063

[6] X. Cao , Y. Wang , C. Wu , et al., “Cathelicidin‐OA1, A Novel Antioxidant Peptide Identified From an Amphibian, Accelerates Skin Wound Healing,” Scientific Reports 8 (2018): 943. 29343843 10.1038/s41598-018-19486-9 PMC5772731

[7] H. Liu , L. Mu , J. Tang , et al., “A Potential Wound Healing‐Promoting Peptide From Frog Skin,” International Journal of Biochemistry & Cell Biology 49 (2014): 32–41. 24441016 10.1016/j.biocel.2014.01.010

[8] H. Liu , Z. Duan , J. Tang , Q. Lv , M. Rong , and R. Lai , “A Short Peptide From Frog Skin Accelerates Diabetic Wound Healing,” FEBS Journal 281 (2014): 4633–4643. 25117795 10.1111/febs.12968

[9] Q.‐Q. Wang , X.‐Q. Lan , X.‐S. Wei , et al., “Amphibian Pore‐forming Protein Βγ‐CAT Drives Metabolite Release From Small Extracellular Vesicles Through Channel Formation,” Zoological Research 44 (2023): 739–742. 37443402 10.24272/j.issn.2095-8137.2022.510 PMC10415774

[10] Y. Wang , Z. Feng , M. F. Yang , et al., “Discovery of a Novel Short Peptide With Efficacy in Accelerating the Healing of Skin Wounds,” Pharmacological Research 163 (2021): 105296. 33220421 10.1016/j.phrs.2020.105296

[11] D. D. Sun , K. Guo , N. X. Liu , et al., “Peptide RL‐QN15 Promotes Wound Healing of Diabetic Foot Ulcers Through p38 Mitogen‐activated Protein Kinase and smad3/miR‐4482‐3p/Vascular Endothelial Growth Factor B Axis,” Burns Trauma 11 (2023): tkad035. 38026443 10.1093/burnst/tkad035 PMC10654477

[12] Y. Wu , Y. Wang , Z. Fu , et al., “Peptide RL‐QN15 Promotes Regeneration of Epidermal Nerve Fibers and Recovery of Sensory Function in Diabetic Skin Wounds,” FASEB Journal 37 (2023): e22892. 36951647 10.1096/fj.202201798RR

[13] P. Qin , Y. Meng , Y. Yang , et al., “Mesoporous polydopamine nanoparticles carrying peptide RL‐QN15 show potential for skin wound therapy,” Journal of Nanobiotechnology 19 (2021): 309. 34627291 10.1186/s12951-021-01051-8 PMC8501717

[14] H. Sun , Y. Wang , T. He , et al., “Hollow polydopamine Nanoparticles Loading With Peptide RL‐QN15: A New Pro‐Regenerative Therapeutic Agent for Skin Wounds,” Journal of Nanobiotechnology 19 (2021): 1–20. 34600530 10.1186/s12951-021-01049-2 PMC8487533

[15] P. Qin , J. Tang , D. Sun , et al., “Zn2+ Cross‐Linked Alginate Carrying Hollow Silica Nanoparticles Loaded With RL‐QN15 Peptides Provides Promising Treatment for Chronic Skin Wounds,” ACS Applied Materials & Interfaces 14 (2022): 29491–29505. 35731847 10.1021/acsami.2c03583

[16] A. Joorabloo and T. Liu , “Recent Advances in Reactive Oxygen Species Scavenging Nanomaterials for Wound Healing,” Exploration 4 (2024): 20230066. 38939866 10.1002/EXP.20230066 PMC11189585

[17] M. Muttenthaler , G. F. King , D. J. Adams , and P. F. Alewood , “Trends in Peptide Drug Discovery,” Nature Reviews Drug Discovery 20 (2021): 309–325. 10.1038/s41573-020-00135-8 33536635

[18] C. Li , Z. Fu , T. Jin , et al., “A Frog Peptide Provides New Strategies for the Intervention Against Skin Wound Healing,” Cellular & Molecular Biology Letters 28 (2023): 61. 37501100 10.1186/s11658-023-00468-3 PMC10375744

[19] C. Li , Y. Xiong , Z. Fu , et al., “The Direct Binding of Bioactive Peptide Andersonin‐W1 to TLR4 Expedites the Healing of Diabetic Skin Wounds,” Cellular & Molecular Biology Letters 29 (2024): 24. 38317065 10.1186/s11658-024-00542-4 PMC10845795

[20] Y. Wang , Y. Li , D. Ni , et al., “miR‐186‐5p Targets TGFβR2 to Inhibit RAW264.7 Cell Migration and Proliferation During Mouse Skin Wound Healing,” Environmental Toxicology 38 (2023): 2826–2835. 37565786 10.1002/tox.23914

[21] Y. Zhang , Y. Wang , L. Zeng , et al., “Amphibian‐Derived Peptide Homodimer OA‐GL17d Promotes Skin Wound Regeneration Through the miR‐663a/TGF‐β1/Smad Axis,” Burns Trauma 10 (2022): tkac032. 35832307 10.1093/burnst/tkac032 PMC9273405

[22] Z. Wei , X. Li , J. Zhou , et al., “Inhibition of miRNA‐365‐2‐5p Targeting SIRT1 Regulates Functions of Keratinocytes to Enhance Wound Healing,” FASEB Journal 39 (2025): 8. 10.1096/fj.202401124RRR 40261275

[23] Y. Li , Z. Fu , C. Deng , et al., “miR‐301a‐5p Regulated IKKβ/NF‐κB Axis and Macrophage Polarization to Accelerate Skin Wound Healing.” International Journal of Biological Macromolecules (2025): 311. 10.1016/j.ijbiomac.2025.143995 40339851

[24] Y.‐T. Wu , Z.‐Q. Ru , Y. Peng , et al., “Peptide Cy RL‐QN15 Accelerates Hair Regeneration in Diabetic Mice by Binding to the Frizzled‐7 Receptor,” Zoological Research 45 (2024): 1287. 39479995 10.24272/j.issn.2095-8137.2024.134 PMC11668943

[25] T. Xiao , Z. Yan , S. X. Xiao , and Y. M. Xia , “Proinflammatory Cytokines Regulate Epidermal Stem Cells in Wound Epithelialization,” Stem Cell Research & Therapy 11 (2020): 232. 32527289 10.1186/s13287-020-01755-y PMC7291661

[26] E. I. Morgun and E. A. Vorotelyak , “Epidermal Stem Cells in Hair Follicle Cycling and Skin Regeneration: A View From the Perspective of Inflammation,” Frontiers in Cell and Developmental Biology 8 (2020): 581697. 33240882 10.3389/fcell.2020.581697 PMC7680886

[27] J. C. Sun , H. Q. Zhao , C. A. Shen , et al., “Tideglusib Promotes Wound Healing in Aged Skin by Activating PI3K/Akt Pathway.” Stem Cell Research & Therapy 13 (2022): 269. 35729652 10.1186/s13287-022-02949-2 PMC9210790

[28] D. Nanba , F. Toki , K. Asakawa , et al., “EGFR‐Mediated Epidermal Stem Cell Motility Drives Skin Regeneration Through COL17A1 Proteolysis,” Journal of Cell Biology 220 (2021): e202012073. 34550317 10.1083/jcb.202012073 PMC8563287

[29] X. D. Chen , R. H. Yang , J. R. Wang , et al., “Porcine Acellular Dermal Matrix Accelerates Wound Healing Through miR‐124‐3p.1 and miR‐139‐5p,” Cytotherapy 22 (2020): 494–502. 32571650 10.1016/j.jcyt.2020.04.042

[30] X. Li , F. Wang , Y. X. Lan , et al., “GDF‐5 Induces Epidermal Stem Cell Migration via RhoA‐MMP9 Signalling,” Journal of Cellular and Molecular Medicine 25 (2021): 1939–1948. 33369147 10.1111/jcmm.15925 PMC7882973

[31] M. Zhang , Y. H. Zhang , J. Ding , et al., “The Role of TrkA in the Promoting Wounding–Healing Effect of CD271 on Epidermal Stem Cells,” Archives of Dermatological Research 310 (2018): 737–750. 30209580 10.1007/s00403-018-1863-3

[32] A. Fakouri , Z. S. Razavi , A. T. Mohammed , A. H. A. Hussein , H. Afkhami , and M. H. Hooshiar , “Applications of Mesenchymal Stem Cell‐Exosome Components in Wound Infection Healing: New Insights,” Burns Trauma 12 (2024): tkae021. 39139205 10.1093/burnst/tkae021 PMC11319788

[33] G. Donati and F. M. Watt , “Stem Cell Heterogeneity and Plasticity in Epithelia,” Cell Stem Cell 16 (2015): 465–476. 25957902 10.1016/j.stem.2015.04.014

[34] S. M. Watt and J. M. Pleat , “Stem Cells, Niches and Scaffolds: Applications to Burns and Wound Care,” Advanced Drug Delivery Reviews 123 (2018): 82–106. 29106911 10.1016/j.addr.2017.10.012

[35] D. Kang , X. Wang , W. Chen , et al., “Epidermal Stem Cell‐derived Exosomes Improve Wound Healing by Promoting the Proliferation and Migration of Human Skin Fibroblasts,” Burns Trauma 12 (2024): tkae047. 39687464 10.1093/burnst/tkae047 PMC11647520

[36] I. J. Augustin , “Wnt Signaling in Skin Homeostasis and Pathology,” Journal Der Deutschen Dermatologischen Gesellschaft 13 (2015): 302–306. 25819237 10.1111/ddg.12620

[37] P. Kaur , “Interfollicular Epidermal Stem Cells: Identification, Challenges, Potential,” Journal of Investigative Dermatology 126 (2006): 1450–1458. 16543901 10.1038/sj.jid.5700184

[38] K. A. U. Gonzales and E. Fuchs , “Skin and Its Regenerative Powers: An Alliance Between Stem Cells and Their Niche,” Developmental Cell 43 (2017): 387–401. 29161590 10.1016/j.devcel.2017.10.001 PMC5797699

[39] M. Sato , “Upregulation of the Wnt/?‐Catenin Pathway Induced by Transforming Growth Factor‐? In Hypertrophic Scars and Keloids,” Acta Dermato‐Venereologica 86 (2006): 300–307. 16874413 10.2340/00015555-0101

[40] P. Huang , R. Yan , X. Zhang , L. Wang , X. Ke , and Y. Qu , “Activating Wnt/β‐Catenin Signaling Pathway for Disease Therapy: Challenges and Opportunities,” Pharmacology & Therapeutics 196 (2019): 79–90. 30468742 10.1016/j.pharmthera.2018.11.008

[41] J. A. McCubrey , D. Rakus , A. Gizak , et al., “Effects of Mutations in Wnt/β‐Catenin, Hedgehog, Notch and PI3K Pathways on GSK‐3 Activity—Diverse Effects on Cell Growth, Metabolism and Cancer,” Biochimica Et Biophysica Acta (BBA)—Molecular Cell Research 1863 (2016): 2942–2976. 27612668 10.1016/j.bbamcr.2016.09.004

[42] C. Y. Janda , L. T. Dang , C. You , et al., “Surrogate Wnt Agonists That Phenocopy Canonical Wnt and β‐catenin Signalling,” Nature 545 (2017): 234–237. 28467818 10.1038/nature22306 PMC5815871

[43] S. Dekoninck and C. Blanpain , “Stem Cell Dynamics, Migration and Plasticity During Wound Healing,” Nature Cell Biology 21 (2019): 18–24. 30602767 10.1038/s41556-018-0237-6 PMC7615151

[44] B. M. Li , H. W. Tang , X. W. Bian , et al., “Calcium Silicate Accelerates Cutaneous Wound Healing With Enhanced Re‐Epithelialization Through EGF/EGFR/ERK‐Mediated Promotion of Epidermal Stem Cell Functions,” Burns Trauma 9 (2021): tkab029. 10.1093/burnst/tkab029 PMC8484206 34604395

[45] T. Xiao , Z. Yan , S. Xiao , and Y. Xia , “Proinflammatory Cytokines Regulate Epidermal Stem Cells in Wound Epithelialization,” Stem Cell Research & Therapy 11 (2020): 232. 10.1186/s13287-020-01755-y PMC7291661 32527289

[46] G. Donati , E. Rognoni , T. Hiratsuka , et al., “Wounding Induces Dedifferentiation of Epidermal Gata6+ Cells and Acquisition of Stem Cell Properties,” Nature Cell Biology 19 (2017): 603–613. 28504705 10.1038/ncb3532 PMC7610974

[47] K. Hoffmeyer , A. Raggioli , S. Rudloff , et al., “Wnt/β‐Catenin Signaling Regulates Telomerase in Stem Cells and Cancer Cells,” Science 336 (2012): 1549–1554. 22723415 10.1126/science.1218370

[48] R. T. Moon , A. D. Kohn , G. V. D. Ferrari , and A. Kaykas , “WNT and β‐Catenin Signalling: Diseases and Therapies,” Nature Reviews Genetics 5 (2004): 691–701. 10.1038/nrg1427 15372092

[49] Y. Shi , B. Shu , R. Yang , et al., “Wnt and Notch Signaling Pathway Involved in Wound Healing by Targeting C‐Myc and Hes1 Separately,” Stem Cell Research & Therapy 6 (2015): 120. 26076648 10.1186/s13287-015-0103-4 PMC4501079

[50] K. Kretzschmar and H. Clevers , “Wnt/β‐Catenin Signaling in Adult Mammalian Epithelial Stem Cells,” Developmental Biology 428 (2017): 273–282. 28526587 10.1016/j.ydbio.2017.05.015

[51] Y. Xi , X. Huang , G. Tan , et al., “Protective Effects of Erdosteine on Interleukin‐1β‐stimulated Inflammation via Inhibiting the Activation of MAPK, NF‐κB, and Wnt/β‐catenin Signaling Pathways in Rat Osteoarthritis,” European Journal of Pharmacology 873 (2020): 172925. 31958453 10.1016/j.ejphar.2020.172925

[52] Y. A. N. Song , X. Qin , H. Wang , et al., “Effects of Integrin α5β1 on the Proliferation and Migration of Human Aortic Vascular Smooth Muscle Cells,” Molecular Medicine Reports 13 (2016): 1147–1155. 26648324 10.3892/mmr.2015.4649 PMC4732837

[53] T. Tsuji , S. Ibaragi , and G.‐f Hu , “Epithelial‐Mesenchymal Transition and Cell Cooperativity in Metastasis,” Cancer Research 69 (2009): 7135–7139. 19738043 10.1158/0008-5472.CAN-09-1618 PMC2760965

[54] H. Sorg , D. J. Tilkorn , S. Hager , J. Hauser , and U. Mirastschijski , “Skin Wound Healing: An Update on the Current Knowledge and Concepts,” European Surgical Research 58 (2017): 81–94. 27974711 10.1159/000454919

[55] P. N. Le , J. D. McDermott , and A. Jimeno , “Targeting the Wnt Pathway in Human Cancers: Therapeutic Targeting With a Focus on OMP‐54F28,” Pharmacology & Therapeutics 146 (2015): 1–11. 25172549 10.1016/j.pharmthera.2014.08.005 PMC4304994

[56] S. Park , D. G. Gonzalez , B. Guirao , et al., “Tissue‐Scale Coordination of Cellular Behaviour Promotes Epidermal Wound Repair in Live Mice,” Nature Cell Biology 19 (2017): 155–163. 28248302 10.1038/ncb3472 PMC5581297

[57] M. Aragona , S. Dekoninck , S. Rulands , et al., “Defining Stem Cell Dynamics and Migration During Wound Healing in Mouse Skin Epidermis,” Nature Communications 8 (2017): 14684. 10.1038/ncomms14684 PMC5339881 28248284

[58] A. Kasuya and Y. Tokura , “Attempts to Accelerate Wound Healing,” Journal of Dermatological Science 76 (2014): 169–172. 25468357 10.1016/j.jdermsci.2014.11.001

[59] A. C. L. Lee , J. L. Harris , K. K. Khanna , and J. H. Hong , “A Comprehensive Review on Current Advances in Peptide Drug Development and Design,” International Journal of Molecular Sciences 20 (2019): 2383. 31091705 10.3390/ijms20102383 PMC6566176

[60] M. A. Aleskandarany , O. H. Negm , A. R. Green , et al., “Epithelial Mesenchymal Transition in Early Invasive Breast Cancer: An Immunohistochemical and Reverse Phase Protein Array Study,” Breast Cancer Research and Treatment 145 (2014): 339–348. 24771047 10.1007/s10549-014-2927-5

[61] T. V. A. Lordani , C. E. de Lara , F. B. P. Ferreira , et al., “Therapeutic Effects of Medicinal Plants on Cutaneous Wound Healing in Humans: A Systematic Review,” Mediators Inflamm 2018 (2018): 1–12. 10.1155/2018/7354250 PMC5901822 29805312

[62] X. Yang , Y. Wang , C. Wu , and E.‐A. Ling , “Animal Venom Peptides as a Treasure Trove for New Therapeutics Against Neurodegenerative Disorders,” Current Medicinal Chemistry 26 (2019): 4749–4774. 30378475 10.2174/0929867325666181031122438

[63] L. Cha , J. Yang , J. Gao , et al., “Bat‐Derived Oligopeptide LE6 Inhibits the Contact–Kinin Pathway and Harbors Anti‐Thromboinflammation and Stroke Potential,” Zoological Research 45 (2024): 1001–1012. 39147715 10.24272/j.issn.2095-8137.2023.372 PMC11491786

[64] S. Yin , A. Pang , C. Liu , et al., “Peptide OM‐LV20 Protects Astrocytes Against Oxidative Stress Via the 'PAC1R/JNK/TPH1'Axis,” Journal of Biological Chemistry 298 (2022): 102429. 36037970 10.1016/j.jbc.2022.102429 PMC9513268

[65] Q. Jia , Z. Fu , Y. Li , et al., “Hydrogel Loaded With Peptide‐Containing Nanocomplexes: Symphonic Cooperation of Photothermal Antimicrobial Nanoparticles and Prohealing Peptides for the Treatment of Infected Wounds,” ACS Applied Materials & Interfaces 16 (2024): 13422–13438. 38442213 10.1021/acsami.3c16061

[66] Y. L. Song , C. Y. Wu , X. H. Zhang , et al., “A Short Peptide Potentially Promotes the Healing of Skin Wound,” Bioscience Reports 39 (2019): BSR20181734. 30842341 10.1042/BSR20181734 PMC6430730

[67] Y. Fu , C. Li , X. J. Li , et al., “Amphibian‐Derived Peptide Homodimer Promotes Regeneration of Skin Wounds,” Biomedicine & Pharmacotherapy 146 (2022): 112539. 34923337 10.1016/j.biopha.2021.112539

[68] R. H. Yang , J. R. Wang , Z. H. Zhou , et al., “Role of Caveolin‐1 in Epidermal Stem Cells During Burn Wound Healing in Rats,” Developmental Biology 445 (2019): 271–279. 30476483 10.1016/j.ydbio.2018.11.015

[69] Y. Zhang , D. Wu , C. Zhou , et al., “Consensus on the Treatment of Second‐Degree Burn Wounds (2024 Edition),” Burns Trauma 12 (2024): tkad061. 10.1093/burnst/tkad061 PMC10858447 38343901

[70] H. Zhang , X. Nie , X. Shi , et al., “Regulatory Mechanisms of the Wnt/β‐Catenin Pathway in Diabetic Cutaneous Ulcers,” Frontiers in Pharmacology 9 (2018): 1114. 30386236 10.3389/fphar.2018.01114 PMC6199358

[71] Q. Li , L. Ye , X. Zhang , et al., “FZD8, a Target of p53, Promotes Bone Metastasis in Prostate Cancer by Activating Canonical Wnt/β‐Catenin Signaling,” Cancer Letters 402 (2017): 166–176. 28602974 10.1016/j.canlet.2017.05.029

[72] W. Zhou , Y. Wang , Y. Huang , et al., “Huangqin Qingre Qubi Capsule Inhibits RA Pathology by Binding FZD8 and Further Inhibiting the Activity of Wnt/β‐Catenin Signaling Pathway,” Journal of Ethnopharmacology 302 (2023): 115886. 36336221 10.1016/j.jep.2022.115886

[73] G. N. Marchenko , N. D. Marchenko , and A. Y. Strongin , “The Structure and Regulation of the Human and Mouse Matrix Metalloproteinase‐21 Gene and Protein,” Biochemical Journal 372 (2003): 503–515. 10.1042/BJ20030174 PMC1223413 12617721

[74] J. Wei , L. Wu , S. Yang , et al., “E‐Cadherin to N‐Cadherin Switching in the TGF‐β1 Mediated Retinal Pigment Epithelial to Mesenchymal Transition,” Experimental Eye Research 220 (2022): 109085. 35500674 10.1016/j.exer.2022.109085

[75] M. Hendricks , Y. Kam , and V. Quaranta , “Cadherin‐Bound β‐Catenin Feeds Into the Wnt Pathway Upon Adherens Junctions Dissociation: Evidence for an Intersection Between β‐Catenin Pools,” PLoS ONE 4 (2009): e4580. 19238201 10.1371/journal.pone.0004580 PMC2640460

[76] S. Y. Sokol , “At the Crossroads Between Cell Polarity and Adhesion in Neocortical Development,” Developmental Cell 41 (2017): 453–454. 28586640 10.1016/j.devcel.2017.05.017

[77] Z.‐Q. Wu , T. Brabletz , E. Fearon , et al., “Canonical Wnt Suppressor, Axin2, Promotes Colon Carcinoma Oncogenic Activity,” Proceedings of the National Academy of Sciences 109 (2012): 11312–11317. 10.1073/pnas.1203015109 PMC3396472 22745173

[78] V. Murillo‐Garzón , I. Gorroño‐Etxebarria , M. Åkerfelt , et al., “Frizzled‐8 Integrates Wnt‐11 and Transforming Growth Factor‐β Signaling in Prostate Cancer,” Nature Communications 9 (2018): 1747. 10.1038/s41467-018-04042-w PMC5931552 29717114

[79] Y. Yang , X. Li , J. Du , Y. Yin , and Y. Li , “Involvement of MicroRNAs‐MMPs‐E‐Cadherin in the Migration and Invasion of Gastric Cancer Cells Infected With Helicobacter Pylori,” Experimental Cell Research 367 (2018): 196–204. 29604247 10.1016/j.yexcr.2018.03.036

[80] T. Ouspenskaia , I. Matos , A. F. Mertz , V. F. Fiore , and E. Fuchs , “WNT‐SHH Antagonism Specifies and Expands Stem Cells Prior to Niche Formation,” Cell 164 (2016): 156–169. 26771489 10.1016/j.cell.2015.11.058 PMC4850916

[81] J. P. Thiery , H. Acloque , R. Y. J. Huang , and M. A. Nieto , “Epithelial‐Mesenchymal Transitions in Development and Disease,” Cell 139 (2009): 871–890. 19945376 10.1016/j.cell.2009.11.007

[82] J. Ding , S.‐J. Lee , L. Vlahos , et al., “Therapeutic Blood‐Brain Barrier Modulation and Stroke Treatment by a Bioengineered FZD4‐Selective WNT Surrogate in Mice,” Nature Communications 14 (2023): 2947. 10.1038/s41467-023-37689-1 PMC10238527 37268690

[83] J. Ma and C. Wu , “Bioactive Inorganic Particles‐Based Biomaterials for Skin Tissue Engineering.” Exploration 2 (2022): 20210083. 37325498 10.1002/EXP.20210083 PMC10190985

[84] G. Du , K. Kataoka , M. Sakaguchi , et al., “Expression of REIC/Dkk‐3 in Normal and Hyperproliferative Epidermis,” Experimental Dermatology 20 (2011): 273–277. 21323747 10.1111/j.1600-0625.2010.01244.x

[85] Q. Feng , S. Xuezhen , L. Haipeng , et al., “Effect of N‐Cadherin on Chondrogenic Differentiation of Bone Marrow‐Derived Mesenchymal Stem Cells Through WNT Signaling Pathway,” Cellular and Molecular Biology 67 (2022): 249–259. 35818189 10.14715/cmb/2021.67.6.33

[86] Z. Zhong and B. O. Williams , “Integration of Cellular Adhesion and WNT Signaling: Interactions Between N‐Cadherin and LRP5 and Their Role in Regulating Bone Mass,” Journal of Bone and Mineral Research 27 (2012): 1849–1851. 22903578 10.1002/jbmr.1715 PMC3904542

[87] Y. Zhang , Y. Xu , H. Kong , et al.,, “Microneedle System for Tissue Engineering and Regenerative Medicine,” Exploration 3 (2023): 20210170. 37323624 10.1002/EXP.20210170 PMC10190997

[88] F. F. Wang , X. Y. Wang , K. Ma , C. P. Zhang , J. Chang , and X. B. Fu , “Akermanite Bioceramic Enhances Wound Healing With Accelerated Reepithelialization by Promoting Proliferation, Migration, and Stemness of Epidermal Cells,” Wound Repair and Regeneration 28 (2020): 16–25. 31270882 10.1111/wrr.12742

