# Supplementary material for: Peptide RL‐QN15 Regulates Functions of Epidermal Stem Cells to Accelerate Skin Wound Regeneration via the FZD8/β‐Catenin Axis
Source: Exploration (Beijing). 2025 Jun 8;6(3):20240090. doi: 10.1002/EXP.20240090 (PMC13317571; doi:10.1002/EXP.20240090)
Supplement: Supplementary file 1 — Supporting File: exp270057‐sup‐0001‐SuppMat.pdf [file EXP2-6-20240090-s001.pdf]

Supporting Information

Peptide RL-QN15 regulates functions of epidermal stem cells to accelerate skin wound regeneration via the FZD8/β-catenin axis

Yuansheng Li[1,#], Qiuye Jia[1,#], Naixin Liu[1], Saige Yin[1], Junyuan Wang[2], Yujing Ding[2], Yuliu Yang[1], Ying Peng[1], Zeqiong Ru[1], Shaoyang Zhang[1], Bu'er Qi[5], Jun Sun[1], Li He[3,*], Ying Wang[2,*] Kun Guo[1,*], Xinwang Yang[1,4*]

1. Department of Anatomy and Histology & Embryology, Faculty of Basic Medical Science, Kunming Medical University, Kunming, 650500, Yunnan, China
2. Key Laboratory of Chemistry in Ethnic Medicinal Resources & Key Laboratory of Natural Products Synthetic Biology of Ethnic Medicinal Endophytes, State Ethnic Affairs Commission & Ministry of Education, School of Ethnic Medicine, Yunnan Minzu University, Kunming, 650504, Yunnan, China
3. Department of Dermatology, First Affiliated Hospital of Kunming Medical University, Kunming, 650032, Yunnan, China
4. Yunnan Yunke Characteristic Plant Extraction Laboratory Co., Ltd., Kunming, 650106, Yunnan, China
5. Department of Restorative Dentistry, Faculty of Dental Medicine, Hokkaido University, Sapporo, 060-8586, Japan.

[#]These authors contributed equally to this work

* Corresponding authors:

Xinwang Yang (yangxinwang@kmmu.edu.cn or yangxinwanghp@163.com),

Kun Guo (kunchong89@163.com);

Ying Wang (wangying_814@163.com)

Li He (drheli2662@ 126.com)

Table S1. Primers used for qRT-PCR

Transcript	Primer sequence (5' to 3')
GAPDH	Sense: AAATCCCATCACCATCTTCCAGG
	Antisense: TGATGACCCTTTTGGCTCCC
CTNNB1	Sense: CTGACCAGCCGACACCAA
	Antisense: ATCAATCCAACAGTAGCCTTTATC
MYC	Sense: TCTGAGGAGGAACAAGAAGATGAGGA
	Antisense: TGAGGACCAGTGGGCTGTGAGG
FZD2	Sense: CCGTGCCGCTCTATCTGTG
	Antisense: GTCCTCGGAGTGGTTCTGGC
FZD8	Sense: AAGGGCATCGGCTACAACTACACCTACAT
	Antisense: TTGAGATCGGGCGAGCACTGGA
LRP1	Sense: ATCCTAATCCCTCTGCTGTTGCTGC
	Antisense: CGTTGGTCATCCGTTGGTGCTG
LRP4	Sense: ACCTTGTATTCTTCAACCACCCG
	Antisense: CATCCCTTTCAGAGCATCTTCCTT
LRP5	Sense: AGCCCGCCCACCACCTTCTT
	Antisense: TCATAGTCGATGGCTTTGACGTTCCT
LRP6	Sense: CAATATCTTGCAGCCTGTGG
	Antisense: TGGACTTTGGTTCTACCCTCT
LRP10	Sense: CCATCCCACCTGTAGAAGACTTTCC
	Antisense: TCCTGGAGTCATATCCTGGCGTAA
LRP11	Sense: TGACCACGCCATCGTCCAGTATGAG
	Antisense: CCTGTAGGTGGGACAGCTTCAGGGTT
MMP3	Sense: TCAGCACTCTGAGGGGAGAA
	Antisense: CATCCACGCCTGAAGGAAGA
MMP10	Sense: TGAGATGCCAGCCAAGTGT
	Antisense: AATGAAATTCAGGTTCAGGGTT

MMP15	Sense:	TGGAACAACCACCATCTGACCTTTAGC
	Antisense:	GGATGTCCTCATAGGGCACCTCCTG
MKI67	Sense:	AAAACGCCCAGGAACACC
	Antisense:	AAGGCTCTGTCTCAGTATCTGAAGT
CDH1	Sense:	GGCGTCTGTAGGAAGGCACA
	Antisense:	TCTCCTCCGAAGAAACAGCAA
CDH2	Sense:	CCAGGTTTGGAATGGGACA
	Antisense:	TTGGGATCGTCAGCATCAA
PCNA	Sense:	GTAATGTCGATAAAGAGGAGGAAG
	Antisense:	ATACTGAGTGTCACCGTTGAAGA
TCF7L1	Sense:	TCAACGAGTCGGAGAACCAGAGC
	Antisense:	TCTCACTTCGGCGAAATAGTCCC
TCF7L2	Sense:	CCCACCACATCATACGCTACA
	Antisense:	GGCTTCTTCTTTTCTTCTTCCTTT
CCND1	Sense:	ATGCCAGAGGCGGAGGAGAACA
	Antisense:	TGGAGGGCGGATTGGAAATGA
ITGA3	Sense:	AGGCACAGGCTCTGGAGAACC
	Antisense:	GCTGCTGCTCTGACACGAAGG
ITGA5	Sense:	ACTCGTCAGACACCCAGGGAACT
	Antisense:	AGATGAGGGACTGTAAACCGAAGG
ITGA6	Sense:	CGAGGTTATGGAACAGCACA
	Antisense:	TTCGGCAGCAGCAGTCA
ITGB1	Sense:	TTGTGAAGCCAGCAACGGACAG
	Antisense:	CAAGGCAGGTCTGACACATCTCAC
LGR4	Sense:	TCCATTGCCTGCTTGCCTGAATC
	Antisense:	GTTCCAGACAACCACCTTGGCTAC

Figure S1

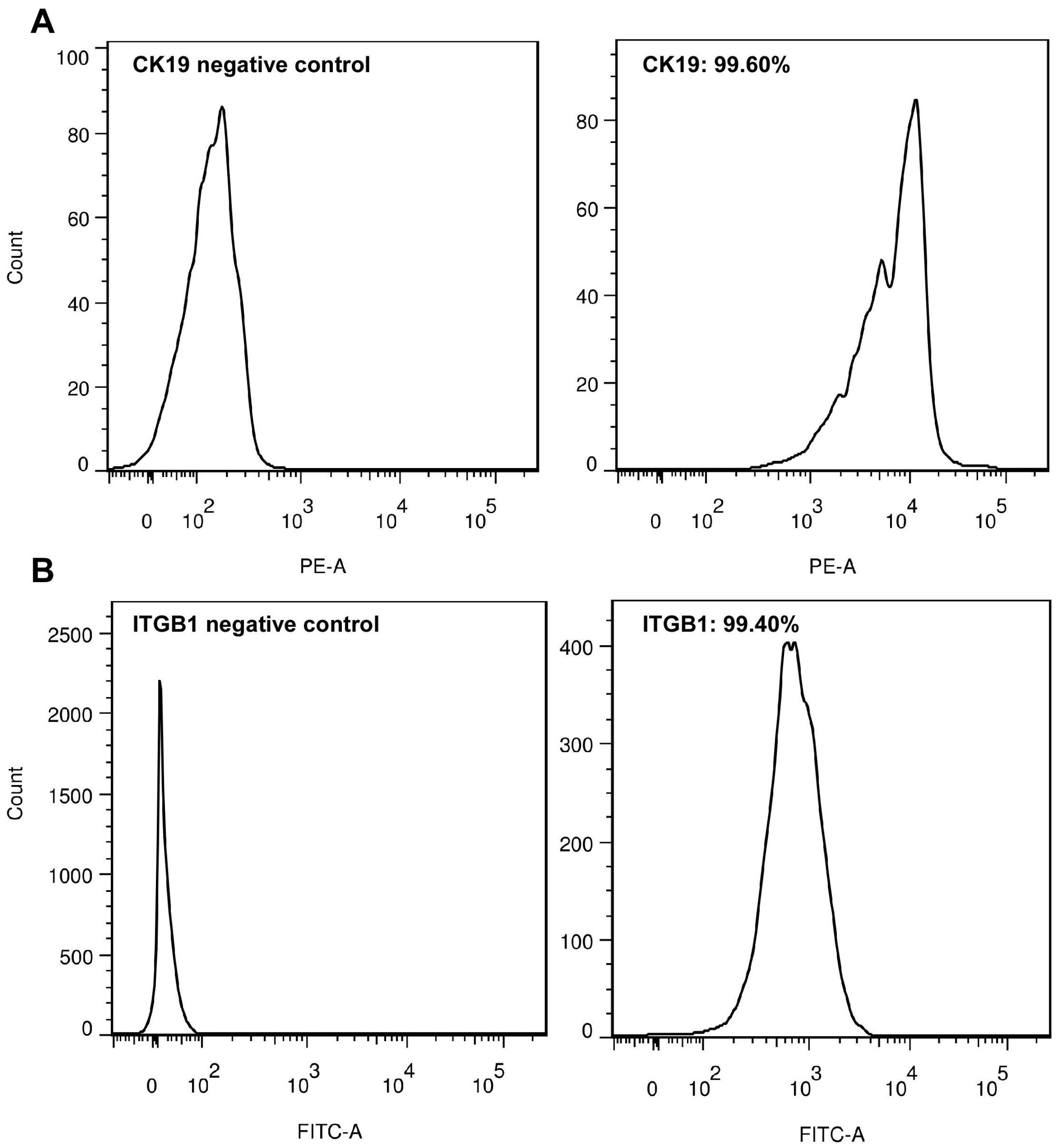

Figure S1. Identification of hESCs by flow cytometry.

(A-B) Flow cytometry analysis revealed that the cultivated cells expressed CK19 and ITGB1, both specific molecular markers for hESCs.

Figure S2

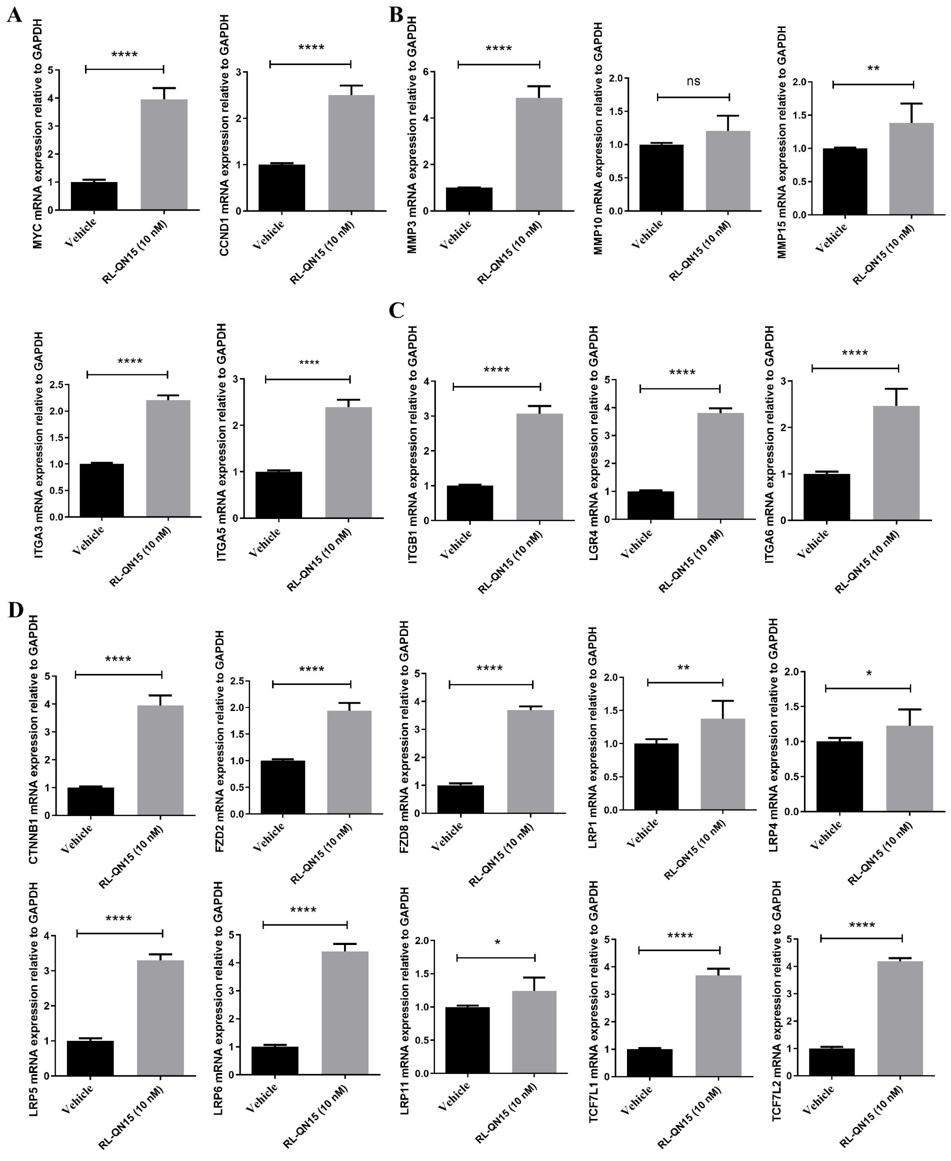

Figure S2. Effects of RL-QN15 on proliferation, migration, stemness-related genes, and the expression of key members of the Wnt/β-catenin signaling pathway in hESCs.

(A-D) Effects of RL-QN15 (10 nM) on the mRNA levels of proliferation-related (*MYC*, *CCND1*), migration-related (*MMP3*, *MMP10*, *MMP15*, *ITGA3*, *ITGA5*), stemness-related genes *(ITGB1*, *LGR4*, *ITGA6*), and key members of the Wnt/β-catenin signaling pathway (*CTNNB1*, *FZD2*, *FZD8*, *LRP1*, *LRP4*, *LRP5*, *LRP6*, *LRP11*, *TCF7L1*, *TCF7L2*) in hESCs, respectively. GAPDH was utilized as an internal reference.

All data were expressed as mean±SD (n=3), *$P < 0.05$, **$P < 0.01$, ***$P < 0.001$, ****$P < 0.0001$. ns, no significance.

Figure S3

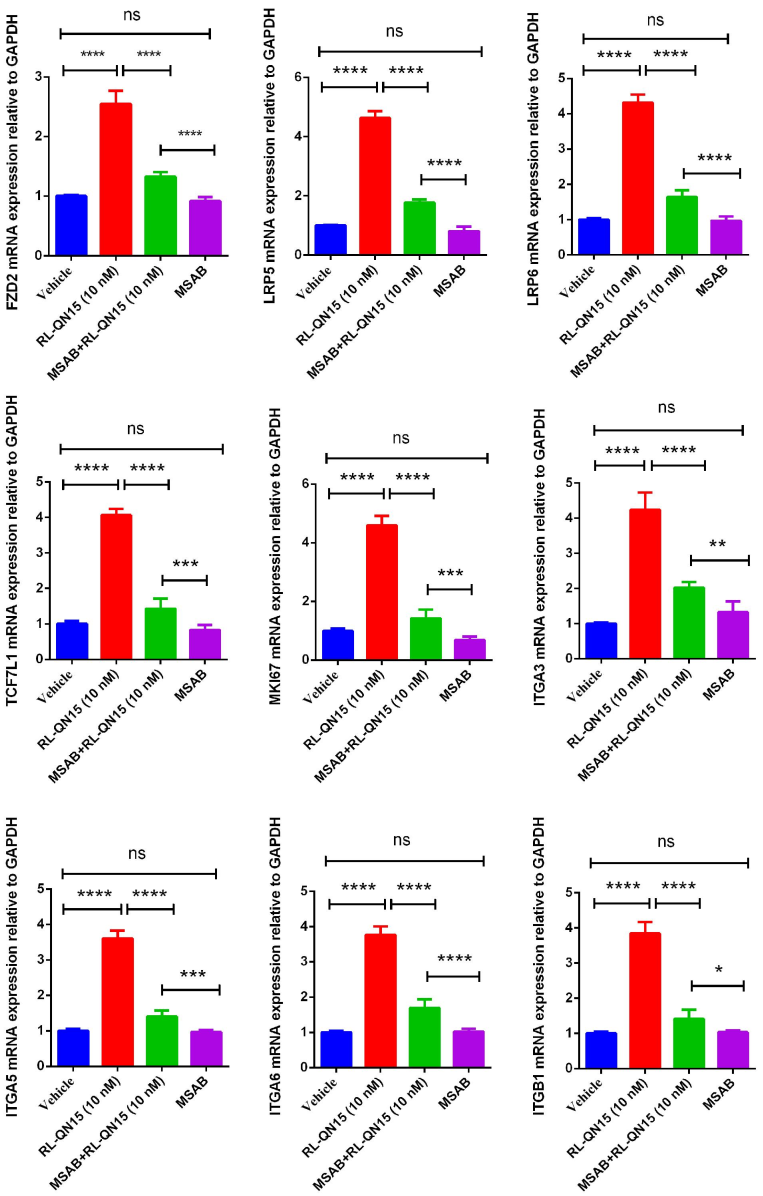

Figure S3. Impacts of MSAB on mRNA levels of Wnt/β-catenin signaling pathway members, proliferation, migration, and stemness related genes in RL-QN15-treated hESCs. Effects of MSAB on the mRNA levels of Wnt/β-catenin signaling pathway components (*FZD2, LRP5, LRP6, TCF7L1*), proliferation-related (*MKI67*), migration-related (*ITGA3,ITGA5* and *ITGA6*) and stemness-related genes (*ITGB1, ITGA6*) in RL-QN15-treated hESCs. GAPDH was used as an internal reference.

All data were expressed as mean±SD (n=3), *P < 0.05, **P < 0.01,***P < 0.001, ****P< 0.0001. ns, no significance.

Figure S4

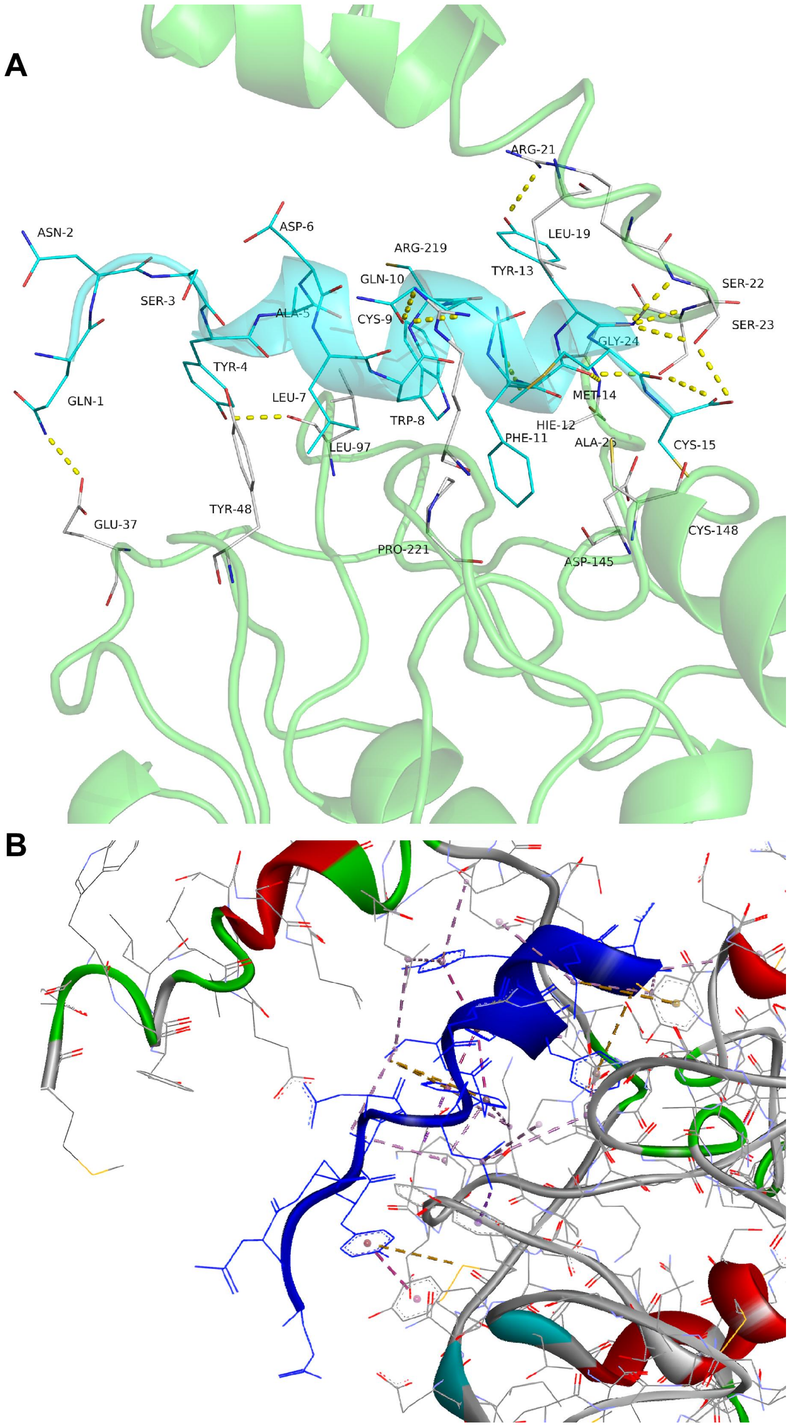

Figure S4. Analysis of molecular docking of RL-QN15 and FZD8.

(A) RL-QN15 contained multiple interacting residues with FZD8, forming multiple hydrogen bonds (yellow dashed lines).

(B) Amino acid residues of RL-QN15 at different sites interact with amino acid residues of FZD8 through aromatic ring-mediated interactions (dashed lines).

Figure S5

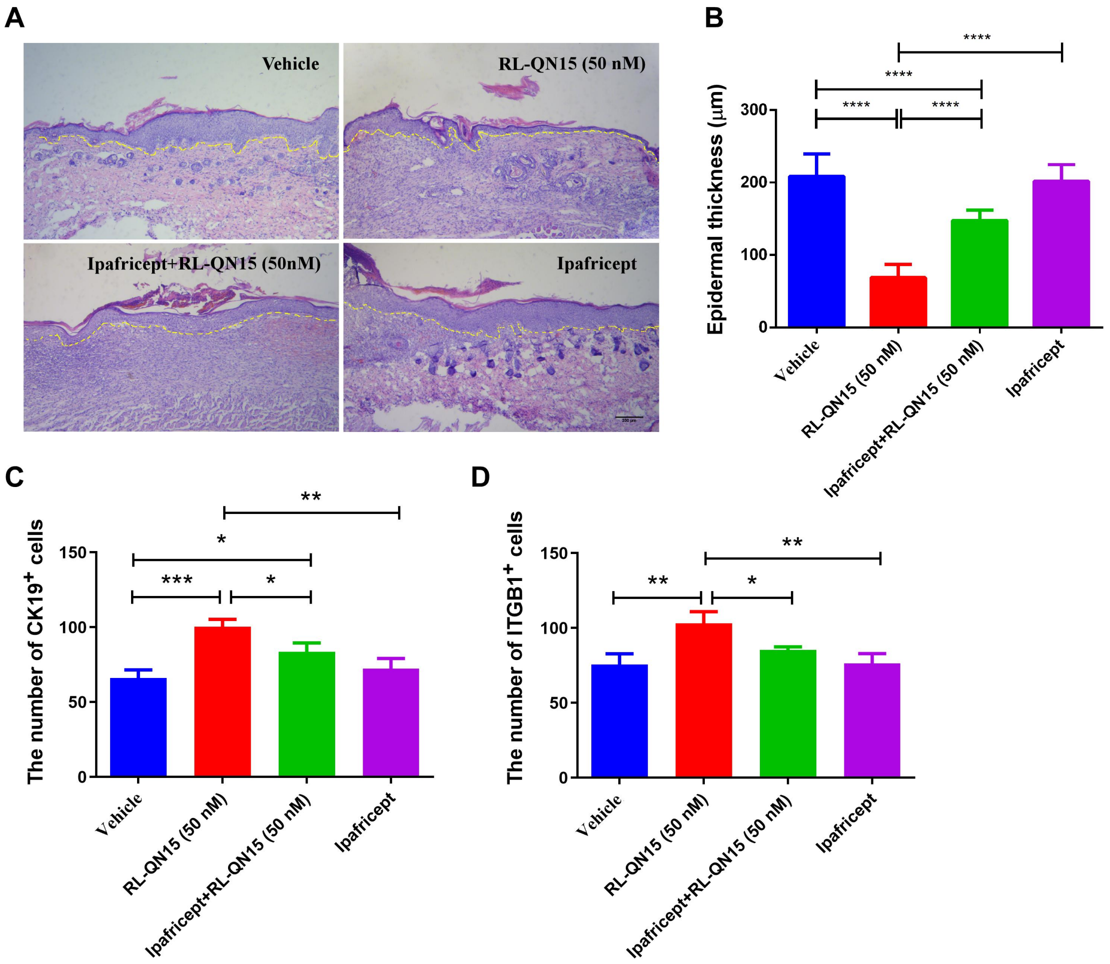

Figure S5 Effect of Ipafricept on neo-epidermal thickness and the number of ESCs in RL-QN15 treated wound skin of mice.

(A) Representative images of H&E staining of mouse wound skin tissues on postoperative day 7 to demonstrate epidermal thickness in each group. Neonatal epidermal boundary was outlined with a yellow dashed line. Scale bar: 200 μm.

(B) Statistical analysis of neo-epidermal thickness in four groups of mice. (*n*=3).

(C-D) The number of ESCs in skin wound tissue from mice in four groups was examined by immunofluorescence staining of frozen sections, with five fields per mouse randomly selected to calculate the number of $CK19^{+}$ and $ITGB1^{+}$ cells in skin wounds. The representative images were showed in Figure 8A, D. (*n*=3).

All data were expressed as mean±SD, $*P < 0.05$, $**P < 0.01$, $***P < 0.001$, $****P < 0.0001$, ns, no significance.
